# CRL4^AMBRA1^-mediated progesterone receptor degradation drives progestin resistance and represents a therapeutic vulnerability in endometrial cancer

**DOI:** 10.7150/ijbs.133072

**Published:** 2026-05-01

**Authors:** Yaru Sheng, Ting Ni, Xiaoyi Ding, Jiaying Huang, Xueya Zhao, Xinyao Li, Qingting Li, Jing Wang, Xiaoming Yang, Lin Zhang, Xuan Zheng, Dan Cao, Rongjia Su, Xiaojing Lu, Jin Hou, Yudong Wang

**Affiliations:** 1 Department of Gynecologic Oncology, The International Peace Maternity and Child Health Hospital, School of Medicine, Shanghai Jiao Tong University, Shanghai, China.; 2 Shanghai Municipal Key Clinical Specialty, Female Tumor Reproductive Specialty, Shanghai, China.; 3 National Key Laboratory of Immunity and Inflammation, Institute of Immunology, Naval Medical University, Shanghai, China

**Keywords:** endometrial cancer, progesterone receptor, ubiquitination modification, medroxyprogesterone acetate resistance

## Abstract

Medroxyprogesterone acetate (MPA) is a major fertility-preserving therapeutic option for patients with endometrial cancer (EC); however, MPA resistance markedly restricts the efficacy of conservative treatments. Downregulation of the progesterone receptor (PR) expression is a key determinant of MPA resistance; however, the underlying molecular mechanisms have not been elucidated comprehensively. In this study, we identified AMBRA1, a substrate-specific adaptor of CUL4-RING E3 ubiquitin ligase (CRL4), as a critical regulator of PR stability and the MPA response. AMBRA1 expression was significantly increased in MPA-resistant EC tissues, and its overexpression induced an MPA-resistant phenotype. Mechanistically, AMBRA1 promotes ubiquitin-mediated degradation of PR by targeting lysine 388 in a CRL4^AMBRA1^ complex-dependent manner. Moreover, pharmacological inhibition of the CRL4^AMBRA1^ complex with MLN4924, an FDA-approved antitumor drug that blocks NEDD8-dependent Cullin-RING ligase activation, stabilizes PR and markedly restores MPA sensitivity in MPA-resistant EC cell lines and patient-derived organoid models. Collectively, these findings suggest that the CRL4^AMBRA1^ ubiquitin ligase facilitates PR degradation, inducing resistance to MPA. Furthermore, this study identified the CRL4^AMBRA1^ complex as a potential therapeutic target for overcoming MPA resistance in EC.

## Introduction

Endometrial cancer (EC) is one of the most prevalent gynecological malignancies worldwide, and its incidence and mortality rates continue to rise^1^. Over the past decade, the incidence has increased by approximately 45%, accompanied by a 5.1% increase in EC-related mortality, with 417,367 new cases and 97,370 deaths reported globally by 2020^2^. Particularly concerning is the steadily growing proportion of reproductive-age patients, which now accounts for up to 14% of all EC cases. More than 70% of these women were nulliparous at the time of diagnosis and expressed a strong desire for future childbearing^3^. Although standard surgical management achieves excellent oncological outcomes in early-stage EC, hysterectomy may compromise fertility preservation, making it unsuitable for young patients who wish to maintain their reproductive potential 4.

In this context, fertility-sparing management of early-stage EC and atypical endometrial hyperplasia in young patients has attracted increasing attention^5^, ^6^. Progestin-based therapy is the first-line fertility-preserving strategy for patients with early-stage low-grade EC who desire to preserve fertility^7^. Medroxyprogesterone acetate (MPA) is the most widely used progestin. Although progestin-based regimens can achieve high initial response rates, de novo and acquired resistance remain major challenges. Approximately 30% of early-stage, low-grade ECs are intrinsically non-responsive to MPA, and up to 57% of initial responders eventually experience recurrence^8^, ^9^, ^10^.

Although progestin has been used clinically for several decades, the mechanisms underlying progestin resistance remain incompletely understood^11^. The effects of progestin are primarily mediated by its binding to the progesterone receptor (PR)^12^. PR expression is critical for maintaining an endocrine-responsive phenotype^7^, and PR levels have been evaluated as potential predictors of the response to progestin therapy in EC^8^, ^13^, ^14^. Progestin binds to the ligand-binding domain (LBD) of PR, particularly the predominant isoform progesterone receptor B (PRB)^15^, activating downstream signaling pathways that regulate multiple PRB-responsive genes involved in apoptosis, differentiation, and cell cycle arrest^16^. Aberrant PR signaling has also been implicated in resistance to sustained progesterone exposure^5^, ^17^, ^18^, and accumulating evidence suggests that restoring PR expression is a key strategy to overcome MPA resistance. As illustrated in EC cell lines, demethylating agents and histone deacetylase inhibitors can enhance MPA sensitivity by upregulating PR^19^, ^20^, ^21^. Chlorpromazine exerts a synergistic effect with MPA by increasing PR expression^22^, and co-administration of neural cell adhesion molecules maintains functional PR and augments MPA sensitivity^23^. However, few of these proposed targets have progressed to the clinical trial stage. Given the complexity of progesterone-mediated molecular alterations in MPA-resistant EC, there is an urgent need to identify critical regulators and delineate the molecular mechanisms that govern PR stability and function.

AMBRA1 (autophagy/Beclin-1 regulator 1) is a vertebrate-conserved, intrinsically disordered adapter protein that was initially characterized as a key positive regulator of autophagy^24^, ^25^. By interacting with Beclin1 to tether the Beclin1-VPS34 complex to the dynein motor complex, and by modulating TRAF6-mediated ubiquitination of ULK1, AMBRA1 promotes autophagy initiation^26^, ^27^, ^28^. AMBRA1 also participates in mitochondrial clearance and apoptosis^29^, ^30^. Recently, dysregulated AMBRA1 has been implicated in tumor progression, including melanoma and breast cancer, through culling-dependent degradation^31^, ^32^, ^33^. Cullin-RING ligases (CRLs) are multi-subunit E3 ubiquitin ligase complexes that orchestrate the proteasomal degradation of multiple substrates, regulating diverse cellular processes. Given their critical roles in tumorigenesis, CRLs have emerged as promising therapeutic targets. MLN4924 (pevonedistat), a potent inhibitor of the NEDD8-activating enzyme, impedes CRLs activity by blocking Cullin neddylation and is being evaluated in clinical trials for its efficacy against various malignancies^34^. However, the role of specific CRL components in EC pathogenesis and the involvement of AMBRA1 in MPA resistance remain largely unknown.

In this study, we identified AMBRA1 as a critical regulator of EC cell responsiveness to progestin therapy. We demonstrated that elevated AMBRA1 expression was associated with MPA resistance in EC, which was validated using *in vitro* and *in vivo* models, as well as clinical samples. Mechanistically, AMBRA1 interacts with PR and promotes its ubiquitination and proteasomal degradation in a CUL4-RING E3 ubiquitin ligase (CRL4)-dependent manner, reducing its stability and leading to MPA resistance. Using patient-derived EC organoids, we further demonstrated that AMBRA1 is a therapeutic target for improving MPA responsiveness and that combined treatment with MPA and the small-molecule NAE inhibitor MLN4924, which targets the CRL4^AMBRA1^ complex, effectively overcame MPA resistance. Collectively, our findings establish that AMBRA1 serves as an important and promising therapeutic target for treating patients with MPA-resistant EC and highlight that targeting the CRL4^AMBRA1^ complex is a promising therapeutic strategy for improving fertility-sparing treatment outcomes.

## Results

### AMBRA1 positively correlated with MPA resistance in endometrial cancer

To identify the key regulators of progestin resistance, we established a paired MPA-resistant cell line by chronically treating MPA-sensitive EC cells with MPA (Supplementary Fig. 1a). Progestin binds to the LBD of its predominant isoform, PRB, activating downstream targets and exerting a suppressive effect on EC cells^16^. After confirming the differential sensitivity to MPA in ISK_Parental and ISK_Res cells (Supplementary Fig. 1b-e), we examined progestin-driven signaling and found that a subset of previously identified PRB-responsive genes was significantly downregulated in ISK_Res cells (Supplementary Fig. 1f). PRB protein levels were substantially reduced in ISK_Res cells compared to those in ISK_Parental cells, whereas *PRB* mRNA levels remained largely unchanged (Supplementary Fig. 1g, h). We hypothesized that other post-translational mechanisms may contribute to PR downregulation in MPA-resistant cells.

**Figure 1:**
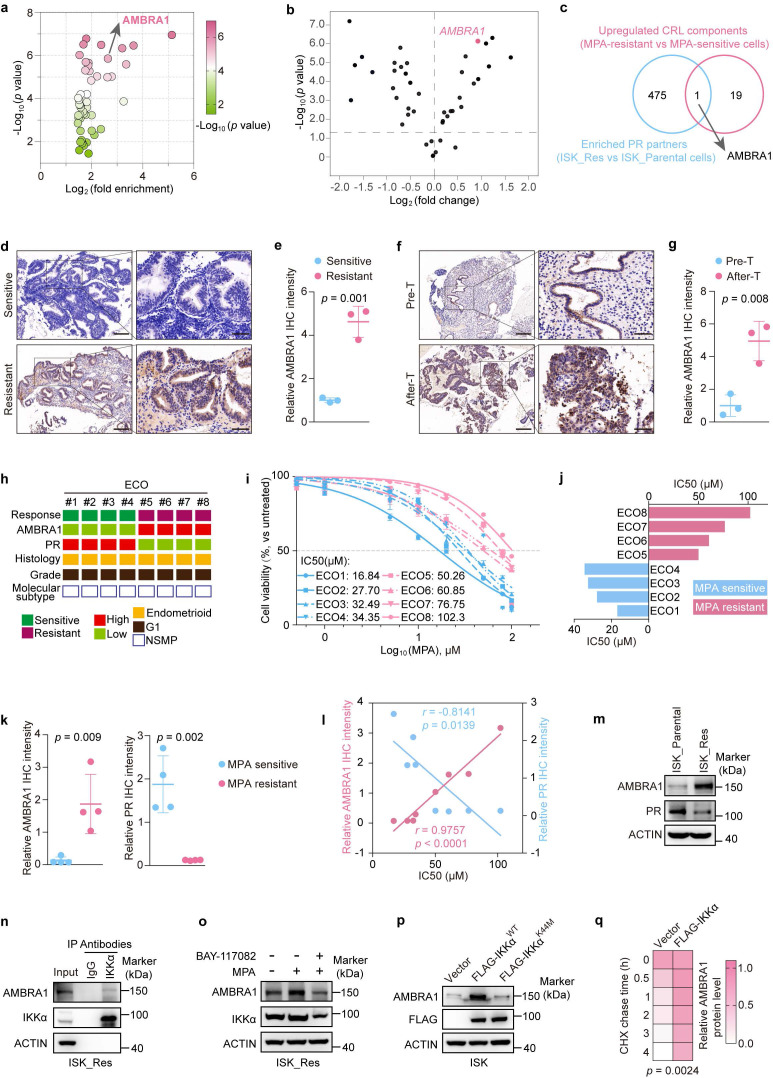
** AMBRA1 is highly expressed in MPA-resistant endometrial cancer and is positively regulated by IKKα. a** A Volcano plot showing the top 50 enriched PR-interacting proteins identified by label-free quantitative proteomics analysis in ISK_Res and ISK_Parental cells. Statistical significance (-Log_10_ (*p-value*)) is indicated by the gradient. **b** Differential expression of Cullin-RING ligase (CRL) components in MPA-resistant and MPA-sensitive EC (data from GSE121367). **c** Venn diagram showing the intersection between PR-interacting proteins enriched in ISK_Res versus ISK_Parental cells (IP-MS) and CRL components upregulated in MPA-resistant versus MPA-sensitive cells. **d** IHC staining of AMBRA1 in post-progestin therapy tumor specimens from patients with MPA-sensitive and MPA-resistant ECs (n = 3 MPA-sensitive samples, n = 3 MPA-resistant samples). Representative images are shown. Scale bars: left panels, 200 μm; right panels, 50 μm. **e** Quantitative analysis of AMBRA1 IHC staining intensity in (**d**). **f** IHC staining images of AMBRA1 in pre-progestin therapy or after therapy tumor specimens from patients with MPA-resistant ECs (n = 3 pre-T samples, n = 3 after-T samples). Representative images are shown. Scale bars: left panels, 200 μm; right panels, 50 μm. Pre-T: pre-progestin therapy; After-T: post-progestin therapy. **g** Quantitative analysis of AMBRA1 IHC staining intensity in (**f**). **h** Characteristics of ECOs from eight patients with EC. Green, MPA-sensitive; dark red, MPA-resistant. AMBRA1 and PR expression levels are low (light green) and high (red) in biopsies. Histology: endometrioids. Grade status: Grade. Molecular subtype: nonspecific molecular profile (NSMP). **i** Dose-response curves for MPA in ECOs. ECOs were cultured at various concentrations of MPA for 6-10 days. Statistical analyses were conducted using an unpaired two-tailed Student's t-test, and the data are presented as the mean ± SD. **j** IC50 of the indicated ECOs against MPA. **k** Quantitative analysis of AMBRA1 and PR IHC staining intensities in MPA-sensitive and MPA-resistant ECOs. **l** Quantitative IHC intensities of AMBRA1 and PR are shown for the eight ECOs. Spearman's correlation analysis was performed to determine the correlation between AMBRA1 and PR with the IC50 of MPA. **m** Western blot analysis of AMBRA1 and PR expression levels in MPA-resistant ISK cells and corresponding parental cells. **n** Endogenous Co-IP assay of the interaction between AMBRA1 and IKKα in ISK_Res cells, and immunoprecipitant was enriched using anti- IKKα antibody, with IgG serving as a negative control. **o** Immunoblotting analysis of AMBRA1 and IKKα expression in ISK_Res cells treated with MPA (20 μM, 48 h) alone or in combination with BAY11-7082 (30 μM, 24h). **p** ISK cells were transiently transfected with either FLAG-IKKα^WT^, FLAG-IKKα^K44M^, or empty vector. Following a 36-hour incubation, the cells were harvested for immunoblotting analysis using anti-AMBRA1 and anti-FLAG antibodies. **q** Quantification of AMBRA1-His protein levels based on band intensity in Supplementary Fig.3**g** is shown. The data in **e**, **g**, and **k** were analyzed using an unpaired two-tailed Student's t-test. Statistical analysis of **q** was performed using a two-way ANOVA with Šídák's correction. The results are presented as the mean ± SD.

To identify post-translational regulators of PR in response to progestin therapy, we performed co-immunoprecipitation followed by 4D label-free quantitative proteomics analysis to profile PR-interacting partners in ISK_Parental and ISK_Res cells. This approach revealed distinct sets of PR-interacting proteins that preferentially associated with PR in ISK_Res cells. AMBRA1 showed a ~6.2-fold increase in enrichment in ISK_Res cells (Fig. 1a). Given the accumulating evidence that CRL components are important therapeutic targets in cancer^35^, we analyzed the transcriptional profiles of CRL components including E3 ligases and adaptor proteins in a published EC dataset^36^ and found that AMBRA1 was ranked as the top differentially expressed gene in MPA-resistant EC compared with MPA-sensitive EC (Fig. 1b, Supplementary Table 1). Subsequently, by integrating our proteomics results with database analysis, we identified AMBRA1 as a high-confidence candidate hit (Fig. 1c). AMBRA1 functions as a substrate-recognition component of the CRL4 ubiquitin E3 ligase complex to control the degradation of multiple proteins^25^, ^37^, ^38^, ^39^ Therefore, we hypothesized that AMBRA1 might modulate PR stability and progestin response in EC. To explore its clinical relevance, we performed AMBRA1 immunohistochemistry (IHC) on human EC specimens that received progestin therapy. Quantitative IHC analysis showed significantly enhanced staining intensity for AMBRA1 in MPA-resistant tumors compared to MPA-sensitive tumors (Fig. 1d, e). Staining of MPA-resistant specimens before and after therapy revealed that AMBRA1 expression was significantly enhanced by progestin treatment (Fig. 1f, g).

We then assessed the role of AMBRA1 in EC and found that AMBRA1 expression was significantly elevated in tumors compared to that in the normal endometrium, as shown by IHC and immunoblotting (Supplementary Fig. 2a-c). In addition, we examined the relationship between AMBRA1 and PR in EC using IHC and observed a negative correlation between AMBRA1 and PR (Supplementary Fig. 2d, e), suggesting that AMBRA1 may regulate MPA sensitivity in EC. No specific molecular profile (NSMP) represents the most prevalent molecular subtype of EC, counting for over 60% of EC cases that receive progestin therapy in clinical practice^40^. Consequently, we generated eight endometrial cancer organoids (ECOs) from surgically resected specimens from patients with NSMP endometrial cancer (Fig. 1h). The cultured ECOs recapitulated the histological features of the corresponding primary tumors, as confirmed by H&E staining (Supplementary Fig. 2f). We assessed AMBRA1 and PR expression in ECOs using IHC and evaluated their sensitivity to MPA (Supplementary Fig. 2g, Fig. 1i, j). We observed a strong positive correlation between higher endogenous AMBRA1 protein levels and higher IC50 values for MPA in eight ECOs samples (r = 0.8987, *p* = 0.0024) (Fig. 1k, l). Moreover, AMBRA1 expression was negatively correlated with PR levels when comparing MPA-sensitive and MPA-resistant ECOs (Fig. 1k, l) and ISK_Parental versus ISK_Res cells (Fig. 1m). Collectively, these data indicate that AMBRA1 is upregulated in MPA-resistant EC and is negatively correlated with PR expression.

### IKKα phosphorylates and stabilizes AMBRA1 upon MPA treatment

We then measured AMBRA1 expression levels between ISK_Parental and ISK_Res cells. In comparison to the modestly elevation observed at the mRNA level (Supplementary Fig. 3a), there was a markedly greater increase at the protein level in ISK_Res cells (Fig. 1m), suggesting that an alteration in protein stability may account for the upregulation of AMBRA1 expression during MPA resistance. IKKα has been reported to stabilize AMBRA1^41^, and immunoprecipitation-mass spectrometry (IP-MS) analysis of AMBRA1-interacting proteins in ISK_Res cells identified IKKα associated with AMBRA1 (Supplementary Fig. 3b). Therefore, we hypothesized that IKKα contributes to the elevated AMBRA1 expression in MPA-resistant cells. Consistently, reciprocal endogenous co-immunoprecipitation (Co-IP) assays showed that AMBRA1 interacted with IKKα in ISK cells, accompanied by increased levels of both proteins in response to MPA. (Supplementary Fig. 3c and d). Endogenous Co-IP further confirmed the association between AMBRA1 and IKKα in ISK_Res cells (Fig. 1n and Supplementary Fig. 3e). Subsequently, we investigated the effects of the IKKα-specific irreversible inhibitor BAY 11-7082 and MPA on the expression of AMBRA1. Consistent with the above results, pharmacological inhibition of IKKα with the irreversible inhibitor BAY 11-7082 largely abrogated MPA-induced AMBRA1 upregulation in both ISK (Supplementary Fig. 3f) and ISK_Res cells (Fig. 1o). Moreover, transient overexpression of wild-type IKKα in ISK cells increased AMBRA1 protein abundance, whereas overexpression of the kinase-dead IKKα^K44M^ mutant had no effect (Fig. 1p). We evaluated the protein stability of AMBRA1 by measuring its half-life in ISK cells treated with the protein synthesis inhibitor cycloheximide (CHX). Compared with the control cells, IKKα overexpression prolonged the half-life of AMBRA1 (Supplementary Fig. 3g and Fig. 1q). Collectively, these data indicate that the activation of IKKα kinase is required for AMBRA1 upregulation during MPA resistance.

Previous research revealed that IKKα phosphorylates AMBRA1 at Ser1043 to regulate mitophagy^41^. Therefore, we examined whether phosphorylation at this site modulates AMBRA1 stability in EC cells. ISK cells were transiently co-transfected with FLAG-IKKα and either His-tagged AMBRA1^WT^ or the phosphorylation-defective AMBRA1^S1043A^ mutant. IKKα overexpression increased the abundance of wild-type AMBRA1 but not that of the S1043A mutant (Supplementary Fig. 3h). Consistently, the AMBRA1^S1043A^ mutant protein exhibited a shorter half-life than wild-type AMBRA1, whereas the kinase-dead IKKα^K44M^ mutant did not affect the wild-type AMBRA1 (Supplementary Fig. 3i, j). These data indicate that IKKα-mediated phosphorylation of AMBRA1 at Ser1043 during MPA treatment stabilizes AMBRA1.

### AMBRA1 promotes MPA resistance in EC

**Figure 2:**
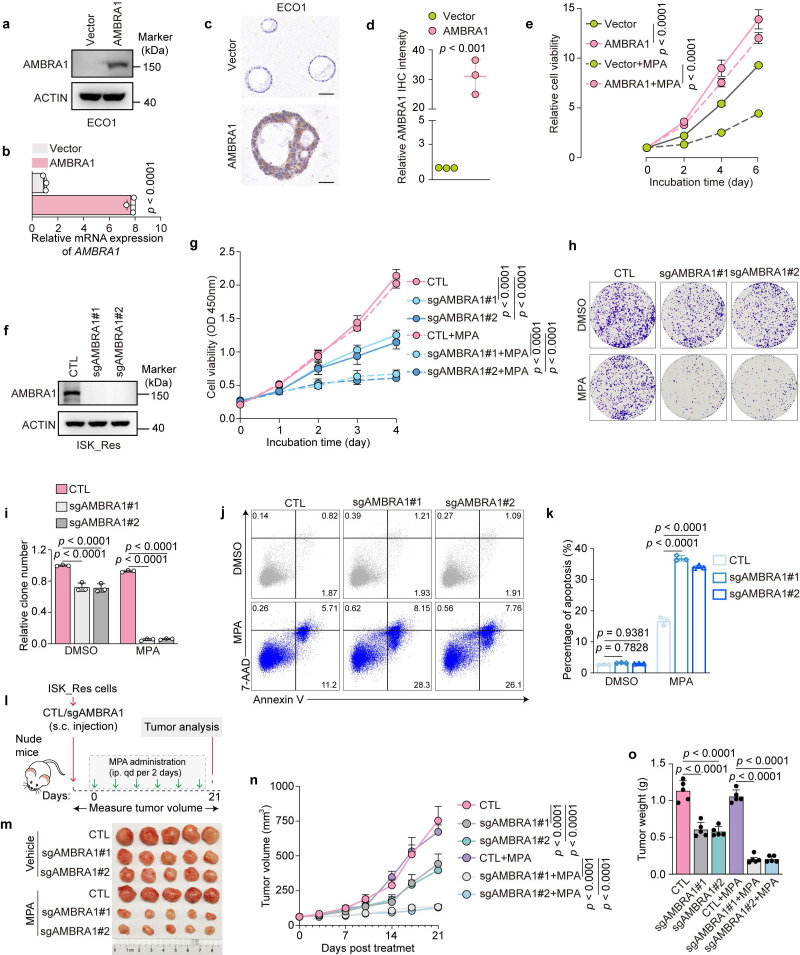
** AMBRA1 regulates MPA sensitivity in EC cells. a**,** b** Overexpression of *AMBRA1* verified by immunoblotting (**a**) and qRT-PCR (**b**) in EC patient-derived organoids (ECO1) (n = three biological replicates per group). **c** Representative IHC staining images of AMBRA1 in ECO1 cells with and without *AMBRA1* overexpression. Scale bars: 50 μm. **d** Quantitative analysis of IHC intensity in (**c**) (n = three biological replicates per group). **e** ECO1 with or without *AMBRA1* overexpression was treated with 20 μM MPA or DMSO for 6 days and then analyzed for cell viability using ATPlite assays (n = three biological replicates per group). **f** Immunoblotting was used to confirm the efficiency of *AMBRA1* knockout in ISK_Res cells. **g** CCK8 assays were performed to detect ISK_Res cells with or without *AMBRA1* knockout in response to 20 μM MPA treatment for 96 hours (n = four biological replicates per group). OD, optical density. **h** Cell growth assay by crystal violet staining of ISK_Res cells with or without *AMBRA1* knockout in response to 20 μM MPA for 10 days. **i** Quantitative analysis of clone numbers in (**h**) (n = three biological replicates per group). **j** CTL and *AMBRA1*-knockout cells were treated with 30 μM MPA or DMSO for 72 h. Apoptosis was detected by flow cytometry after staining with Annexin V and 7-AAD. **k** Percentage of apoptotic cells in (**j**) (n = three biological replicates per group). **l** Diagram depicting the *in vivo* growth of tumor xenografts by subcutaneous inoculation with ISK_Res cells, as generated in (**f**). **m** Xenograft tumors derived from ISK_Res cells with or without *AMBRA1* knockout in response to MPA treatment. **n**, **o** Monitored tumor volume (**n**) and final weight (**o**) are shown (n = 5). Data in **b** and **d** were analyzed using an unpaired two-tailed Student's t-test. Statistical analyses for **i**,** k**, and **o** were performed using one-way ANOVA with Šídák's correction. Statistical analyses of **e**, **g**, and **n** were performed using a two-way ANOVA with Šídák's correction. All results are presented as the mean ± SD.

To explore the role of AMBRA1 in the regulation of MPA responsiveness, we first generated an AMBRA1-overexpressing model in the MPA-sensitive EC cell line ISK (Supplementary Fig. 4a, b). AMBRA1 overexpression markedly blunted MPA-induced growth inhibition, as evidenced by increased cell proliferation and colony formation compared to control cells treated with MPA (Supplementary Fig. 4c-e), and a concomitant reduction in apoptosis (Supplementary Fig. 4f, g). Next, we overexpressed AMBRA1 in MPA-sensitive organoid ECO1 (Fig. 2a-d). As expected, AMBRA1 overexpression attenuated MPA-induced cell viability in ECO1 cells (Fig. 2e). Subsequently, we established xenograft tumors derived from ISK cells with or without AMBRA1 overexpression and treated them with MPA (Supplementary Fig. 4h). AMBRA1 overexpression markedly decreased the sensitivity of ISK xenografts to MPA treatment compared to the control group, as indicated by limited tumor growth (Supplementary Fig. 4i) and reduced tumor volume and weight (Supplementary Fig. 4j, k).

To further validate AMBRA1 as an effective target to overcome MPA resistance in EC, we established *AMBRA1* knockout (KO) ISK_Res cells using CRISPR-Cas9 (Fig. 2f). AMBRA1 KO significantly increased the sensitivity of ISK_Res cells to MPA, resulting in reduced cell viability and colony formation after MPA treatment (Fig. 2g-i), and significantly increased MPA-induced apoptosis compared to control cells (Fig. 2j, k). Finally, we subcutaneously injected AMBRA1-depleted and control ISK_Res cells into the flanks of nude mice, which were further treated with MPA (Fig. 2l). Consistent with the *in vitro* results, AMBRA1 KO dramatically suppressed tumor growth under MPA resistance and reduced tumor volume and weight, enhancing the *in vivo* efficacy of MPA (Fig. 2m-o). Taken together, these results demonstrate that AMBRA1 promotes MPA resistance in EC.

### AMBRA1 interacts with PR and attenuates progestin signaling

AMBRA1 has been reported to promote autophagy^24^, ^25^; however, using the specific autophagy activator rapamycin and autophagy inhibitor chloroquine (CQ), we did not observe reproducible changes in autophagy flux upon AMBRA1 overexpression in ISK cells, with or without MPA treatment (Supplementary Fig. 5a, b). We performed a luciferase reporter assay to investigate the mechanism underlying AMBRA1-mediated MPA resistance. RANKL, a direct downstream target of PR suppressed by PR upon MPA treatment, showed markedly increased promoter-driven luciferase activity after MPA treatment in AMBRA1-overexpressing ISK cells compared to that in control cells (Supplementary Fig. 5c). Consistently, RANKL protein levels were not efficiently inhibited by MPA in AMBRA1-overexpressing ISK cells (Supplementary Fig. 5d). qRT-PCR further demonstrated that AMBRA1 overexpression markedly reduced MPA-induced upregulation of PRB-responsive genes (Supplementary Fig. 5e, f). Taken together, these data indicate that AMBRA1 substantially attenuates progestin signaling in EC cells.

**Figure 3:**
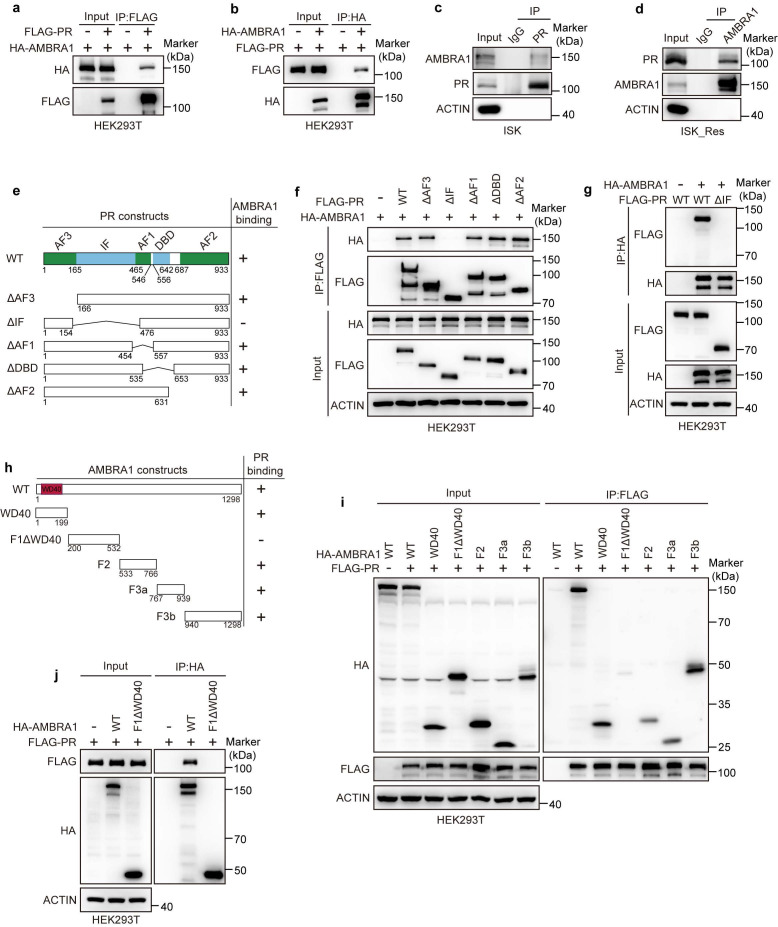
** AMBRA1 interacts with PR. a**, **b** Exogenous Co-IP assay of the interaction between FLAG-PR and HA-AMBRA1 in HEK293T cells. FLAG-PR and HA-AMBRA1 were overexpressed in HEK293T cells, and immunoprecipitates were enriched with anti-FLAG (**a**) or anti-HA (**b**) antibody. **c**, **d** Reciprocal endogenous Co-IP assay of the interaction between AMBRA1 and PR in ISK and ISK_Res cells. After MG132 treatment (20 μM, 6 h), immunoprecipitants were enriched with anti-PR (**c**) or anti-AMBRA1 (**d**) antibody. IgG served as the negative control. **e** Schematic representation of PR wild-type (WT) and truncated mutants. **f**, **g** FLAG-PR WT or truncated mutants were co-expressed with HA-AMBRA1 in HEK293T cells. After 36h cells were harvested for immunoprecipitation with FLAG (**f**) or HA (**g**) beads, followed by immunoblotting. **h** Schematic depiction of AMBRA1 WT and its different truncates. **i**, **j** HA-AMBRA1 WT or the indicated truncation mutants were co-expressed with FLAG-PR in HEK293T cells. After 36h cells were harvested for immunoprecipitation with FLAG (**i**) or HA (**j**) beads, followed by immunoblotting.

To explore how elevated AMBRA1 dampens progestin signaling, we first verified whether AMBRA1 interacts with PR, as suggested by our proteomics analysis (Fig. 1a). We first found and confirmed using exogenous Co-IP assays in HEK293T cells ectopically expressing FLAG-PR and HA-AMBRA1 that AMBRA1 was associated with PR (Fig. 3a), and reciprocal Co-IP further confirmed this interaction (Fig. 3b). The interactions between endogenous AMBRA1 and PR were further confirmed in ISK and ISK_Res cells using endogenous Co-IP assays (Fig. 3c, d). To further delineate the structural determinants mediating AMBRA1-PR binding, we generated a series of truncated mutants of both proteins. Co-IP analysis using PR deletion mutants revealed that the IF domain of PR was required for its interaction with AMBRA1 (Fig. 3e, f), which was confirmed by reciprocal Co-IP analysis (Fig. 3g). Mapping experiments on AMBRA1 showed that both the N-terminal WD40 domain (aa 1-199) and the C-terminal region (F2, F3a, F3b; aa 533-1298) bound PR with an affinity comparable to that of full-length AMBRA1, whereas the fragment encompassing amino acids 200-532 (F1ΔN-WD40) failed to interact with PR (Fig. 3h, i). Reciprocal Co-IP confirmed these results (Fig. 3j). Collectively, these data demonstrate that AMBRA1 interacts with PR and attenuates progestin signaling in EC.

### AMBRA1 downregulates PR protein level by ubiquitin-mediated degradation in EC cells

**Figure 4:**
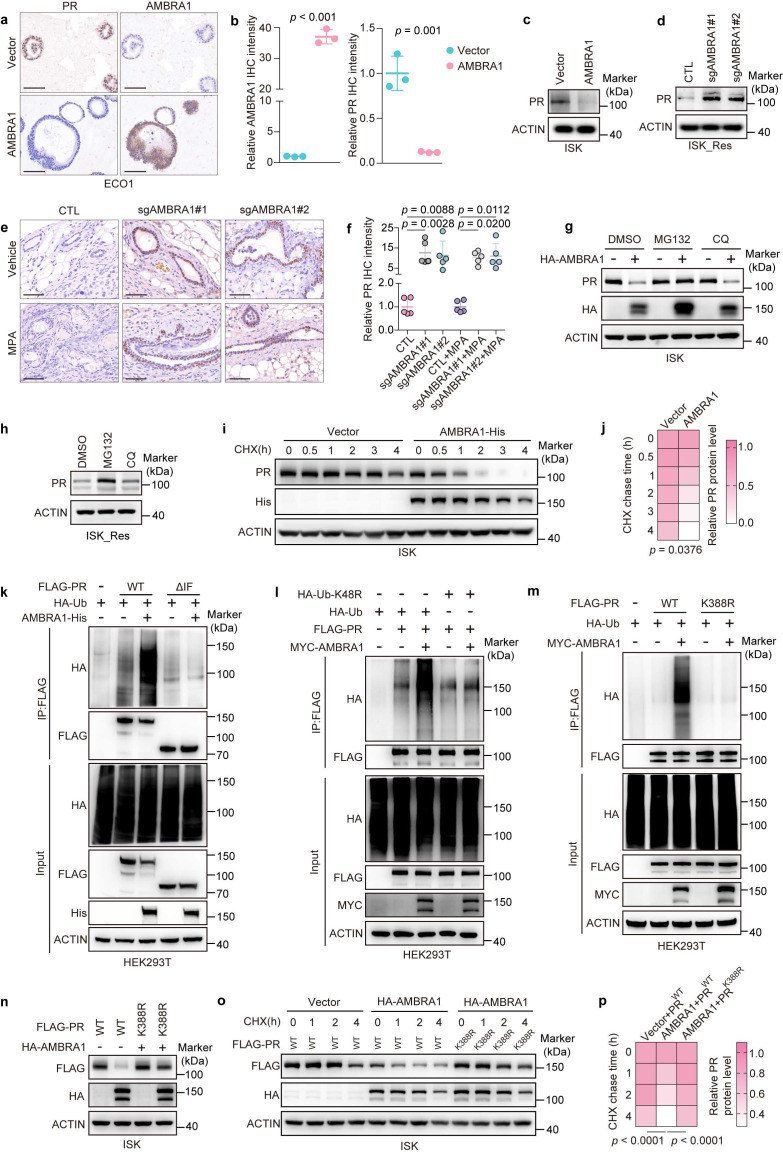
** AMBRA1 promotes the ubiquitin-mediated degradation of PR at lysine 388. a** Representative IHC staining images of PR and AMBRA1 in ECO1 cells with or without *AMBRA1* overexpression. Scale bars: 100 μm. **b** Quantitative analysis of IHC intensity in (**a**) (n = three biological replicates per group). **c** Immunoblotting analysis of PR protein level in *AMBRA1-overexpressed* ISK cells. **d** Immunoblotting analysis of PR protein level in *AMBRA1*- knockout ISK_Res cells. **e** Representative images of IHC staining of PR in xenograft tumors from ISK_Res cells with *AMBRA1* knockout. Scale bars: 50 μm. **f** Quantitative analysis of PR IHC staining intensity in (**e**). **g** Immunoblotting analysis of PR and HA-AMBRA1 expression in AMBRA1-overexpressing ISK cells after treatment with MG132 (20 μM, 6 h) or CQ (40 μM, 12 h). **h** Immunoblotting analysis of PR expression in ISK_Res cells after treatment with MG132 (20 μM, 6 h) or CQ (40 μM, 12 h). **i** Immunoblotting analysis of PR and AMBRA1 in AMBRA1-overexpressing ISK cells treated with or without cycloheximide (CHX, 50 μg/mL) for the indicated time. **j** Quantification of PR protein levels based on band intensity (n = three biological replicates). **k** AMBRA1-His and HA-ubiquitin (HA-Ub) were co-expressed with FLAG-PR WT or ΔIF mutant in HEK293T cells. After MG132 (20 μM, 6 h) treatment, Co-IP was performed with FLAG beads, followed by immunoblotting with the indicated antibodies. **l** MYC-AMBRA1 and WT FLAG-PR were co-expressed with HA-Ub or mutant ubiquitin (HA-Ub-K48R) in HEK293T cells. After MG132 (20 μM, 6 h) treatment, Co-IP was performed with FLAG beads, followed by immunoblotting with the indicated antibodies. **m** MYC-AMBRA1 and HA-Ub were co-expressed with WT FLAG-PR or its K388R mutant in HEK293T cells. After MG132 (20 μM, 6 h) treatment, Co-IP was performed with FLAG beads, followed by immunoblotting with the indicated antibodies. **n** HA-AMBRA1 was co-expressed with FLAG-PR WT or K388R plasmids in ISK cells, followed by immunoblotting with the indicated antibodies. **o** HA-AMBRA1 was co-transfected with the FLAG-PR WT or K388R plasmid into ISK cells and treated with or without cycloheximide (CHX, 50 μg/mL) for the indicated time. Immunoblotting was performed for FLAG-PR and HA-AMBRA1. **p** Quantification of FLAG-PR protein levels based on band intensity (n = three biological replicates per group). Data in **b** were analyzed using an unpaired two-tailed Student's t-test. Statistical analyses for **f** were performed using one-way ANOVA with Šídák's correction. Statistical analyses of **j** and **p** were performed using two-way ANOVA with Šídák's correction. All results are presented as the mean ± SD.

As a substrate-recognition subunit of the CRL4^AMBRA1^ ubiquitin ligase, we hypothesized that AMBRA1 binds to PR and downregulates PR protein levels via ubiquitin-proteasome degradation. To examine the relationship between AMBRA1 and PR expression, we first performed IHC staining on AMBRA1-overexpressing ECO1 organoids. Quantitative analysis revealed that AMBRA1-overexpressing organoids displayed markedly weaker PR staining than control organoids (Fig. 4a, b). Consistently, stable AMBRA1 overexpression substantially reduced PR protein levels in ISK cells (Fig. 4c), whereas AMBRA1 KO ISK_Res cells showed increased PR protein levels (Fig. 4d). IHC analysis of xenograft tumor tissues further confirmed that ablation of AMBRA1 significantly upregulated PR expression in vivo (Fig. 4e, f). Neither AMBRA1 overexpression nor AMBRA1 KO altered PR mRNA levels in EC cells or organoids (Supplementary Fig. 5g), suggesting that AMBRA1 regulates PR at the post-transcriptional level.

To determine whether this regulation depends on the ubiquitin-proteasome system, we ectopically expressed AMBRA1 in ISK cells and treated them with either the proteasome inhibitor MG132 or the lysosomal inhibitor CQ. AMBRA1 overexpression reduced endogenous PR protein levels, whereas MG132, but not CQ, restored PR expression and largely reversed the AMBRA1-induced decrease in PR levels (Fig. 4g). Consistent with this, MG132, but not CQ, increased PR protein levels in ISK_Res cells (Fig. 4h). Next, we assessed PR stability by monitoring its decay following CHX treatment. Compared with control cells, AMBRA1-overexpressing ISK cells exhibited a markedly shortened PR half-life (Fig. 4i, j), indicating that AMBRA1 promotes PR destabilization via proteasomal degradation.

We tested whether AMBRA1 promotes the ubiquitination of PR. His-tagged AMBRA1, HA-ubiquitin (HA-Ub), and either wild-type (WT) or ΔIF mutant FLAG-PR (impaired AMBRA1 binding) were co-expressed in HEK293T cells. AMBRA1 robustly enhanced ubiquitination of WT PR (Fig. 4k). The ΔIF mutant displayed only a minimal increase in ubiquitination upon AMBRA1 expression, consistent with its reduced ability to bind to AMBRA1 (Fig. 4k and Fig. 3f, g). To determine the type of ubiquitin linkage involved, we co-transfected plasmids expressing AMBRA1 with HA-tagged WT ubiquitin or single-lysine-changed Ub mutants (K6R, K11R, K27R, K29R, K33R, K48R, and K63R). This analysis revealed that AMBRA1 predominantly mediates K48-linked polyubiquitination of PR (Supplementary Fig. 6a and Fig. 4l). Collectively, these results demonstrate that AMBRA1 promotes K48-linked polyubiquitination and proteasomal degradation of PR in EC cells.

To identify the lysine acceptor of PR in AMBRA1-mediated ubiquitin conjugation, we generated a panel of 34 PR mutants in which lysine residues were individually or in combination substituted with arginine, collectively covering all 41 lysine residues in PR. The AMBRA1-mediated ubiquitination of these PR variants was examined in HEK293T cells. *In vivo* ubiquitination assays revealed that the double mutant K386R+K388R of PR, in which lysines 386 and 388 were mutated to Arg, was the only mutant that could not be effectively ubiquitylated in the presence of AMBRA1 (Supplementary Fig. 6b-d), suggesting that these two lysines are potential AMBRA1-mediated ubiquitination sites for PR. To further define the major site, we individually mutated lysine 386 or lysine 388 to arginine and performed *in vivo* ubiquitination assays. As shown in Supplementary Fig. 6e and Fig. 4m, exogenous overexpression of AMBRA1 in HEK293T cells increased the ubiquitination of WT FLAG-PR. This increase in PR ubiquitination was also observed in the K386R mutant of FLAG-PR but was almost completely abolished in the K388R mutant, validating that lysine 388 of PR is the primary ubiquitylation site required for AMBRA1-mediated ubiquitination. Moreover, overexpression of HA-AMBRA1 led to an apparent decrease in the level of WT PR, but did not affect the K388R mutant (Fig. 4n). In addition, the K388R mutant of PR protein exhibited a longer half-life than WT PR (Fig. 4o, p).

Collectively, these findings indicate that AMBRA1 mediates K48-linked ubiquitination of PR, with lysine 388 being the predominant ubiquitination site targeted by AMBRA1 to regulate MPA responsiveness in EC cells.

### CRL4^AMBRA1^ complex is required for AMBRA1-mediated PR ubiquitination

**Figure 5:**
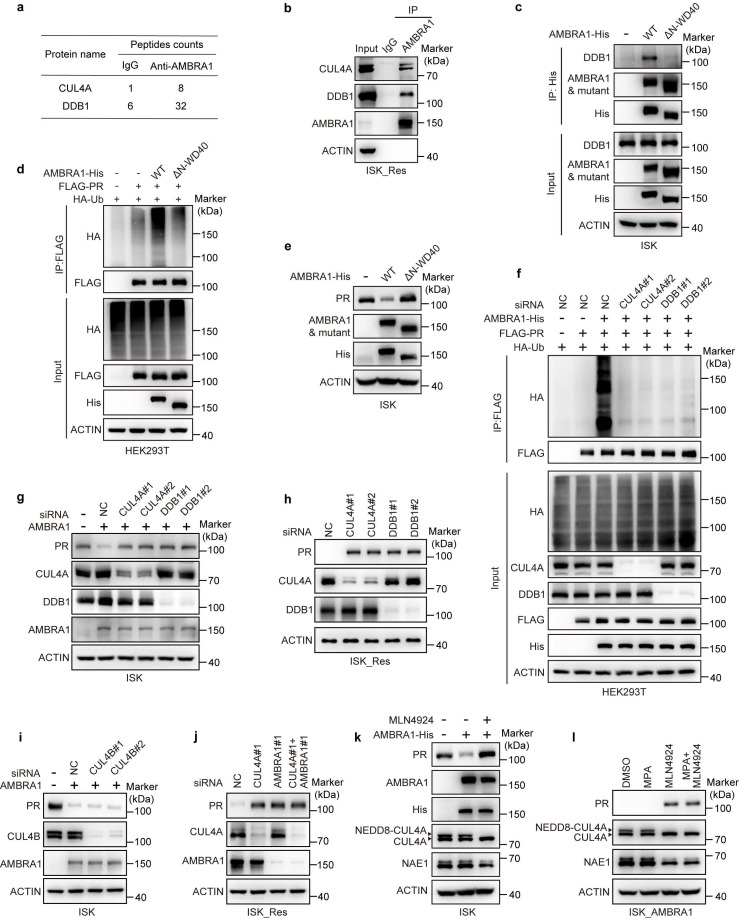
** AMBRA1 promotes PR ubiquitylation in a CRL4-dependent manner in EC cells. a** Table showing the CRL4 core components interacting with AMBRA1 in ISK_Res cells. **b** Endogenous Co-IP assays of the interactions between AMBRA1 and CRL4 core members CUL4A and DDB1 in ISK_Res cells. Immunoprecipitates were enriched with anti-AMBRA1 antibody. IgG served as the negative control. **c** ISK cells were transiently transfected with AMBRA1-His WT or the ΔN-WD40 mutant. After transfection for 48 h, Co-IP was performed using an anti-His antibody, followed by immunoblotting using the indicated antibodies. **d** FLAG-PR and HA-Ub were co-expressed with AMBRA1-His WT or ΔN-WD40 mutants in HEK293T cells. After MG132 (20 μM, 6 h) treatment, Co-IP was performed with FLAG beads, followed by immunoblotting with the indicated antibodies. **e** ISK cells were transiently transfected with AMBRA1-His WT or ΔN-WD40 mutant, and after 48 h, protein levels were examined by immunoblotting analysis with appropriate antibodies, as indicated. **f** Immunoassay for PR ubiquitination in HEK293T cells following AMBRA1 overexpression and *CUL4A* or *DDB1* knockdown using siRNAs. Cells were pretreated with 20 μM MG132 for 6 h. **g** Immunoblotting analysis of PR in *AMBRA1-overexpressing* ISK cells following *CUL4A* or *DDB1* knockdown using siRNAs. **h** Immunoblotting analysis of PR in ISK_Res cells following *CUL4A* or *DDB1* knockdown using siRNAs. **i** Immunoblot analysis of PR in* AMBRA1*-overexpressing ISK cells following *CUL4B* knockdown using siRNAs. **j** Immunoblotting analysis of PR in ISK_Res cells following *CUL4A* and / or *AMBRA1* knockdown using siRNAs. **k** Immunoblot analysis of PR, neddylated-CUL4A, and NAE1 in ISK cells with exogenous AMBRA1 overexpression following treatment with MLN4924 (0.1 μM, 8 h). **l** Immunoblotting assays were performed to detect the expression levels of PR, neddylated-CUL4A, and NAE1 in ISK cells with stable AMBRA1 overexpression after treatment with MPA (20 μM, 24h) or / and MLN4924 (0.1 μM, 8h).

AMBRA1 belongs to the DDB1- and CUL4-associated factor (DCAF) family, which specifies substrates for CRL4 ubiquitin ligase complex^25^, ^37^, ^38^, ^39^. AMBRA1 also functions as an essential cofactor for the RING finger E3 ubiquitin ligase TRAF6, which regulates the ubiquitination of its substrates^42^. To elucidate the mechanism of AMBRA1-mediated PR ubiquitination, we performed IP-MS to identify AMBRA1-interacting proteins in EC cells. The results showed that the CRL4 core components CUL4A and DDB1 were associated with AMBRA1, whereas TRAF6 was not (Fig. 5a). We speculate that the ligase activity of the CRL4^AMBRA1^ complex is required to regulate PR protein levels. We first confirmed the interaction between AMBRA1 with CUL4A and DDB1, the two core subunits of CRL4 ubiquitin ligases, using endogenous Co-IP in ISK_Res cells (Fig. 5b). Because the N-terminal region containing a WD40 domain has been reported to mediate DDB1 binding and is critical for the assembly of the CRL4^AMBRA1^ ubiquitin ligase complex^33^, we constructed an AMBRA1 mutant by deleting the N-terminal region (ΔN-WD40, aa 1-179). Subsequently, semi-endogenous Co-IP of exogenous WT or ΔN-WD40 AMBRA1 with endogenous DDB1 in ISK cells showed that, unlike WT AMBRA1, the ΔN-WD40 mutant lost its DDB1-binding capability (Fig. 5c) and failed to increase PR ubiquitination (Fig. 5d). Consistent with this, ectopic expression of the ΔN-WD40 mutant did not affect PR protein abundance, whereas full-length AMBRA1 markedly reduced PR levels in ISK cells (Fig. 5e), suggesting that AMBRA1-mediated regulation of PR expression depends on the CRL4^AMBRA1^ ubiquitin ligase complex.

In addition, knockdown of endogenous DDB1 or CUL4A by siRNA significantly reduced AMBRA1-driven PR ubiquitylation in 293T cells (Fig. 5f). In ISK cells stably overexpressing AMBRA1, silencing CUL4A or DDB1 markedly blunted the AMBRA1-induced decrease in PR protein levels (Fig. 5g). Consistently, silencing of CUL4A or DDB1 in ISK_Res cells robustly increased PR protein abundance (Fig. 5h). In contrast, silencing CUL4B failed to reverse the AMBRA1-mediated reduction in PR protein levels (Fig. 5i). Moreover, individual knockdown of either CUL4A or AMBRA1, as well as combined kncokdown of both CUL4A and AMBRA1 in ISK_Res cells, all strongly increased PR protein abundance (Fig. 5j), indicating that CUL4A plays a specific role in AMBRA1-mediated PR downregulation. MLN4924 is a selective inhibitor of the NEDD8-activating enzyme, which is required for CRL activity^34^. Therefore, we assessed whether pharmacological inhibition of CRL4 modulates AMBRA1-mediated PR regulation and MPA response. After confirming that MLN4924 effectively inhibited NAE1 expression and reduced CUL4A neddylation, we observed that MLN4924 treatment reversed the AMBRA1-induced reduction of PR protein levels in ISK cells (Fig. 5k) and increased PR protein levels in ISK cells with stable AMBRA1 overexpression (Fig. 5l). Collectively, these findings indicate that AMBRA1 downregulates PR predominantly by acting as a substrate receptor for the CRL4^AMBRA1^ ubiquitin ligase complex.

### Targeting the CRL4^AMBRA1^ complex reverses AMBRA1-induced MPA resistance

**Figure 6:**
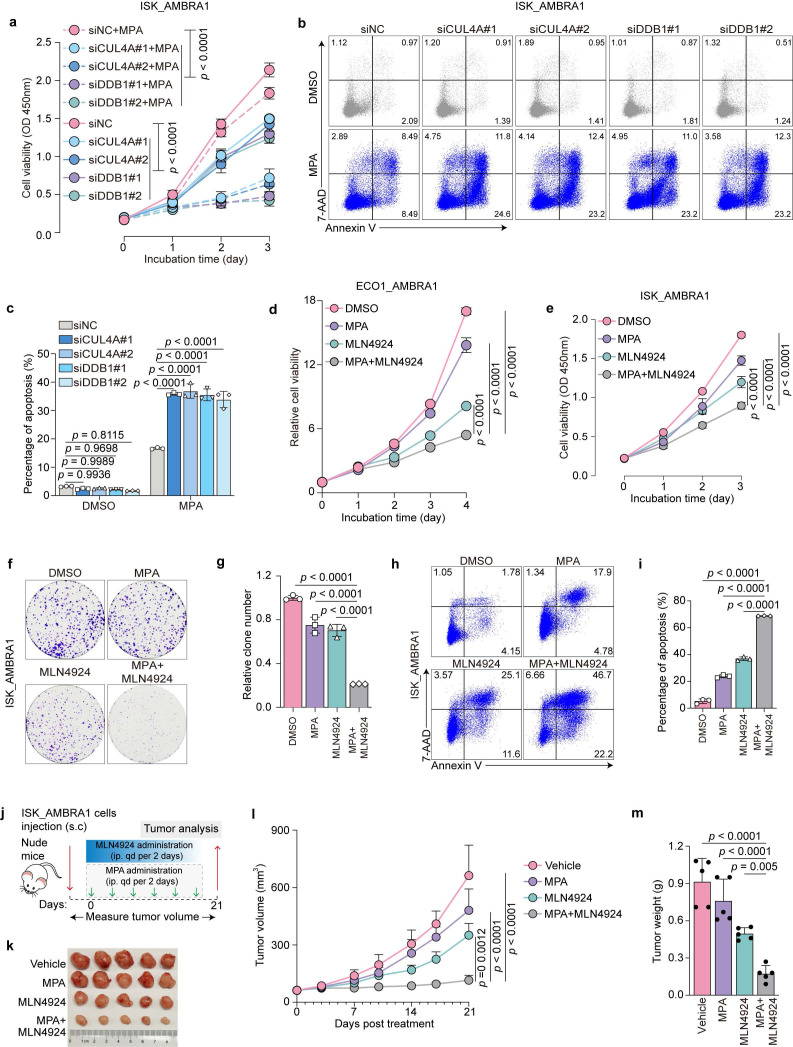
** Targeting the CRL4 complex attenuates AMBRA1-driven MPA resistance in EC. a** CCK8 assays were performed to detect *AMBRA1* overexpressed ISK cells with or without *CUL4A* or* DDB1* knockdown in response to 20 μM MPA treatment for 72 h (n = three biological replicates per group). OD, optical density. **b**, **c** siRNA transfected *AMBRA1* overexpressed ISK cells were treated with DMSO or 30μM MPA for 72 h. Apoptosis was detected by flow cytometry after staining with Annexin V and 7-AAD. The percentage of apoptotic cells is shown in (**c**) (n = three biological replicates per group). **d** *AMBRA1* overexpressed ECO1 were treated with MPA (20 μM), MLN4924 (0.1 μM), or a combination of both for the indicated days and then analyzed for cell viability using the ATPlite assay (n = three biological replicates per group). **e** CCK8 assays were performed to detect the combined effect of MPA (20 μM) treatment and MLN4924 (0.1 μM) in *AMBRA1*-overexpressing ISK cells. OD, optical density (n = four biological replicates per group). **f** Cell growth assay by crystal violet staining of *AMBRA1*-overexpressing ISK cells treated with MPA (20 μM), MLN4924 (0.05 μM), or a combination for 10 days. **g** Quantitative analysis of the clone numbers in (**f**) (n = three biological replicates per group). **h** *AMBRA1*-overexpressing ISK cells were treated with MPA (20 μM) and/or MLN4924 (0.1 μM) for 72 h. Apoptosis was detected by flow cytometry after staining with Annexin V and 7-AAD. **i** Percentage of apoptotic cells in (**h**) (n = three biological replicates per group). **j** Diagram depicting the *in vivo* growth of tumor xenografts after subcutaneous inoculation with *AMBRA1*-overexpressing ISK cells. **k** Xenograft tumors derived from *AMBRA1*-overexpressing ISK cells in response to MPA, MLN4924, or a combination of treatments. **l**, **m** Monitored tumor volume (**l**) and weight (**m**) are shown (n = 5). Statistical analyses in **a**,** d**,** e**, and** l** were performed using a two-way ANOVA with Šídák's correction. Data in **c**,** g**,** i**, and** m** were analyzed using one-way ANOVA with Šídák's correction. All results are presented as the mean ± SD.

Because AMBRA1 overexpression confers MPA resistance in EC cells by promoting CRL4^AMBRA1^-dependent PR degradation, we hypothesized that inhibiting the CRL4^AMBRA1^ complex would restore MPA sensitivity. Therefore, we individually knocked down CUL4A or DDB1 in AMBRA1-overexpressing ISK cells using siRNAs and confirmed their efficient depletion by immunoblotting (Fig. 5g). CUL4A and DDB1 knockdown markedly reversed MPA resistance caused by AMBRA1 overexpression, as evidenced by a reduced cell survival rate in cell viability assays upon MPA treatment (Fig. 6a) and an increased percentage of apoptotic cells, as measured by flow cytometry (Fig. 6b, c). Consistently, immunoblotting results showed that the NEDD8-activating enzyme inhibitor, MLN4924, significantly restored PR protein levels, which were reduced by AMBRA1 overexpression in ISK cells (Fig. 5j), suggesting that MLN4924 is a potential therapeutic agent for inhibiting CRL4 activity in MPA-resistant EC. Next, we examined whether MLN4924 could restore MPA sensitivity in AMBRA1-overexpressing EC models and found that MLN4924 markedly alleviated AMBRA1-induced MPA resistance in patient-derived organoids (ECO1), as reflected by a decrease in cell viability (Fig. 6d). Similar effects were observed in AMBRA1-overexpressing ISK cells, as shown by the reduced cell viability (Fig. 6e), decreased colony formation (Fig. 6f, g), and enhanced apoptosis upon MLN4924 treatment (Fig. 6h, i). In line with the *in vitro* findings, MLN4924 effectively abrogated AMBRA1-induced MPA resistance in AMBRA1-overexpressing ISK xenografts *in vivo* (Fig. 6j), as indicated by attenuated tumor growth (Fig. 6k) and reduced tumor volume and weight (Fig. 6l, m). Together, these results identify the CRL4^AMBRA1^ complex as a critical mediator of MPA resistance in EC and indicate that targeting the CRL4^AMBRA1^ complex may be a promising strategy to overcome MPA resistance.

### Pharmacological inhibition of the CRL4^AMBRA1^ complex sensitizes endometrial cancer to progestin therapy

**Figure 7:**
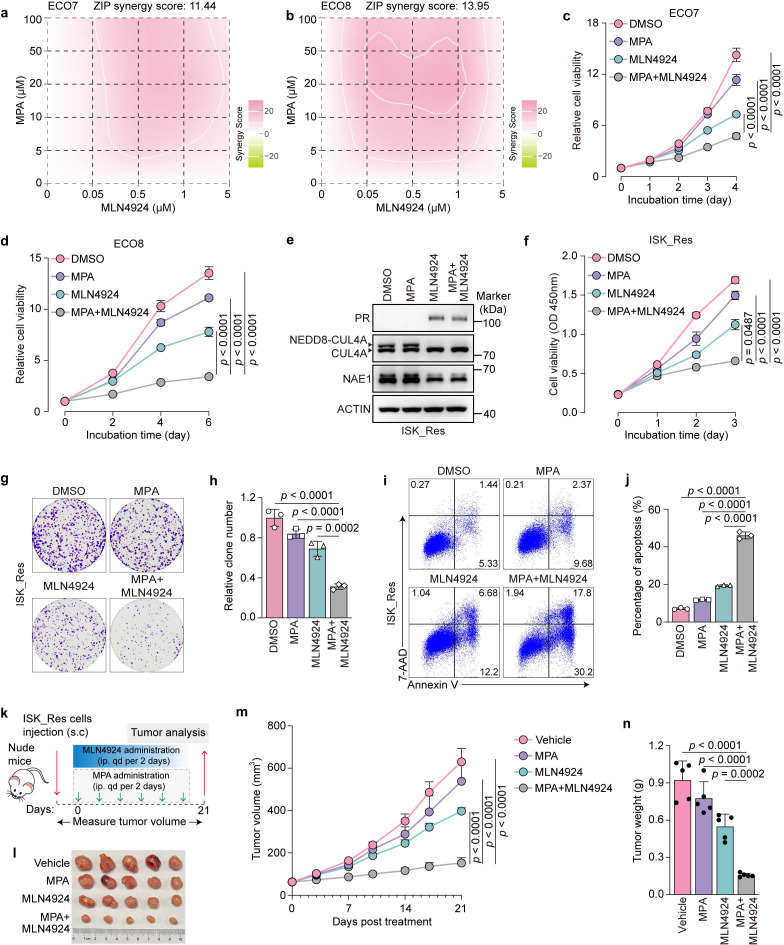
** MLN4924 synergizes with MPA to suppress the growth of MPA-resistant EC cells and ECOs. a**, **b** Indicated ECOs were treated as indicated for 6-10 days and cell viability was analyzed using the ATPlite assay. Synergy graphs and scores were generated using SynergyFinder (ZIP model). **c**, **d** Indicated ECOs were treated with MPA (20 μM), MLN4924 (0.1 μM), or a combination for 6-10 days, followed by an assessment of cell viability using the ATPlite assay (n = three biological replicates per group). **e** Immunoblotting assays were performed to detect the expression levels of PR, neddylated-CUL4A, and NAE1 in ISK_Res cells after treatment with MPA (20 μM, 24h) or / and MLN4924 (0.1 μM, 8h). **f** CCK8 assays were performed to detect the combination effect of MPA (20 μM) and MLN4924 (0.1 μM) in ISK_Res cells (n = 4 biological replicates per group). OD, optical density. **g** Cell growth assay by crystal violet staining of ISK_Res cells treated with MPA (20 μM), MLN4924 (0.05 μM), or a combination for 10 days. **h** Quantitative analysis of clone numbers in **f** (n = 3 biological replicates per group). **i** ISK_Res cells were treated with MPA (20 μM), MLN4924 (0.1 μM), or a combination of both for 72 h. Apoptosis was detected by flow cytometry after staining with Annexin V and 7-AAD. **j** Percentage of apoptotic cells at **h** (n = 3 biological replicates per group). **k** Diagram depicting the *in vivo* growth of tumor xenografts by subcutaneous inoculation with ISK_Res cells. **l** Xenograft tumors derived from ISK_Res cells in response to MPA, MLN4924, or a combination of both. **m**, **n** Monitored tumor volume (**m**) and weight (n) are shown (n = 5). Statistical analyses in (**c**), (**d**), (**f**), and (**m**) were performed using two-way ANOVA with Šídák's correction. The data in (**h**), (**j**), and (**n**) were analyzed using one-way ANOVA with Šídák's correction. All results are presented as the mean ± SD.

We investigated the potential synergistic antitumor effects of MLN4924 and MPA in MPA-resistant EC models. Patient-derived ECO7 and ECO8 organoids were treated with MPA alone or in combination with MLN4924. The results demonstrated that MPA exhibited a considerable synergistic effect with MLN4924, as indicated by synergy scores of 11.44 and 13.95, respectively (Fig. 7a, b). Based on these synergistic effects, we selected the minimum effective concentrations of MLN4924 and MPA for subsequent combination assays. The combination with MLN4924 markedly enhanced MPA sensitivity in ECO7 and ECO8 organoids, as evidenced by a pronounced decrease in cell viability (Fig. 7c, d). Moreover, MLN4924 significantly enhanced the ability of MPA to induce PRB-responsive gene expression (Supplementary Fig. 7a, b). Consistent with these findings, similar combinatorial effects were observed in MPA-resistant ISK_Res cells, as demonstrated by the restored PR expression (Fig. 7e), markedly reduced cell survival (Fig. 7f), decreased colony formation (Fig. 7g, h), and increased apoptosis (Fig. 7i, j) following treatment with MPA plus MLN4924. We evaluated the therapeutic efficacy of this combination *in vivo* (Fig. 7k). While the control tumors expanded rapidly despite MPA treatment and MLN4924 alone demonstrated limited therapeutic efficacy, the combination of MPA and MLN4924 substantially restored the sensitivity to MPA, resulting in significantly suppressed tumor growth (Fig. 7l-n). Collectively, these results indicate that pharmacological inhibition of the CRL4^AMBRA1^ complex enhances the sensitivity of EC cells to progestin therapy.

## Discussion

The frequency of EC among nulliparous women is increasing, and a growing number of women of reproductive age are delaying childbearing, creating an increasing need for effective fertility-sparing strategies^1^, ^2^, ^3^. High-dose progestins, particularly MPA, are the current standard conservative treatments; however, primary or acquired progestin resistance is common and remains a major therapeutic dilemma^8^, ^10^. In this study, we identified AMBRA1 as a key determinant of MPA responsiveness in EC. We showed that AMBRA1 regulates PR stability in a CRL4^AMBRA1^ -dependent manner and that pharmacological inhibition of CRL4 activity with the NEDD8-activating enzyme inhibitor MLN4924 stabilizes PR and restores MPA sensitivity in EC cell lines, patient-derived organoids, and *in vivo* models. These findings reveal a mechanism by which MPA resistance is controlled by the CRL4^AMBRA1^ -PR axis and suggest that targeting this complex may improve the efficacy of progestin therapy.

Several strategies have been proposed to overcome progesterone resistance by restoring PR expression or function, including DNA demethylating agents^21^, histone deacetylase inhibitors^19^, ^20^, and other epigenetic or signaling modulators^22^, ^23^. Although these approaches can increase PR transcription or enhance PR signaling, their therapeutic benefits are limited^7^. Ube3c has been reported to regulate PR protein stability in the endometrium and modulate progesterone responsiveness during early pregnancy^43^. Similarly, SOX4 can regulate decidualization of human endometrial stromal cells by promoting PR stability, which provides valuable insights into the pathology of decidualization-related infertility and may lay the foundation for improving pregnancy outcomes^44^. However, the post-transcriptional regulation of PR in MPA-resistant EC has not been sufficiently explored.

Our study complements these efforts by delineating a post-translational mechanism that directly controls PR protein turnover, as supported by the following evidence: (1) AMBRA1 specifically interacts with PR; (2) upregulation of AMBRA1 accelerates PR turnover; (3) AMBRA1 overexpression promotes K48-linkage ubiquitination of PR; and (4) lysine 388 of PR is the predominant ubiquitylation attachment site catalyzed by AMBRA1. Lysine 388 within the IF domain has been reported as a key site, which is not only involved in PR SUMOylation but also a critical site for CUEDC2-mediated ubiquitination in breast cancer^45^. Consistent with the role of PR as a transcription factor, we showed that AMBRA1 also attenuated MPA-responsive gene expression. Our study delineates a mechanism by which PR is governed by AMBRA1 at the post-translational level and provides in-depth insights into the regulation of AMBRA1 on progestin responsiveness in EC.

AMBRA1 is a scaffold protein characterized by intrinsically disordered regions, conferring high structural plasticity and the ability to engage in multiple protein-protein interactions. Early studies characterized AMBRA1 as a positive regulator of the autophagy signaling network; its dysregulation or genetic mutation results in neurological disorders in mouse models and humans^46^, ^47^. AMBRA1 also participates in apoptosis and orchestrates the balance between autophagy and cell death^48^, influencing cell fate and drug sensitivity^49^, ^50^. Recently, AMBRA1 dysregulation has been implicated in several cancers, including melanoma^32^, breast cancer^33^, prostate cancer^51^, and pancreatic cancer^52^, often via ubiquitination-mediated proteasomal degradation of critical substrates. These observations highlight the context-dependent role of AMBRA1 in different genetic backgrounds and tumor microenvironments. Our study extends these findings to EC by demonstrating that AMBRA1 promotes MPA resistance and identifies PR as a direct substrate. We demonstrated that AMBRA1 binds to and ubiquitinates PR, attenuating progestin signaling and conferring MPA resistance in an autophagy-independent manner.

CRLs represent the largest subclass of E3 ligases and are increasingly being viewed as attractive therapeutic targets^33^. MLN4924, an inhibitor of the NEDD8-activating enzyme, blocks culling neddylation and broadly suppresses CRL activity, and has been tested in clinical trials for several malignancies^34^, ^53^, ^54^, ^55^, ^56^. In this study, we demonstrated that AMBRA1-mediated ubiquitination of PR requires the CRL4^AMBRA1^ complex and that MLN4924 effectively reverses MPA resistance by restoring PR levels *in vitro*, in patient-derived organoids, and in xenograft models. Although MLN4924 is not selective for CRL4^AMBRA1^, our genetic evidence, including CUL4A and DDB1 knockdown and disruption of the AMBRA1-DDB1 binding domain, strongly implicates CRL4^AMBRA1^ as the principal mechanistic target. AMBRA1 contains numerous intrinsically disordered regions, and effective druggable strategies based on conventiaonal drug design remain unavailable to date. Given that siRNA-based agents are suitable for intrauterine delivery^57^, employing AMBRA1-specific siRNA therapy to disrupt the assembly of the CRL4^AMBRA1^ complex may also extend the therapeutic window of fertility-sparing treatments for patients with EC in future investigations.

In conclusion, our study uncovered a critical role for the CRL4^AMBRA1^ ubiquitin ligase in controlling PR stability and progestin responsiveness in EC. By demonstrating that AMBRA1-dependent PR degradation promotes MPA resistance and that pharmacological inhibition of CRL4^AMBRA1^ with MLN4924 restores MPA sensitivity, we provide evidence that AMBRA1 and the CRL4^AMBRA1^ complex are promising targets for improving the efficacy of fertility-preserving progestin therapy in women with EC.

## Methods

### Study approval

All animal studies were conducted in accordance with the National Institutes of Health Guide for the Care and Use of Laboratory Animals and were approved by the Ethics Committee of the International Peace Maternity and Child Health Hospital, China (Approval Number: GKDW-A-2024-55). Organoid culture, immunohistochemistry, and immunoblotting of human EC tissues were approved by the Ethics Committee of the International Peace Maternity and Child Health Hospital, China (Approval Number: GKLW-A-2025-130-01) in accordance with the principles of the Declaration of Helsinki. Clinical samples were collected at the International Peace Maternity and Child Health Hospital, and informed consent was obtained from each participant. None of the patients had other reproductive system diseases. Pathological diagnosis of EC or endometrial hyperplasia was made according to the latest National Comprehensive Cancer Network (NCCN) guidelines. All patients received MPA for at least 6 months and were regularly followed up. Patients were defined as MPA-sensitive if no residual hyperplasia or carcinoma was detected in > 95% of the tissue. Patients were classified as MPA-resistant if less than 50% of the hyperplastic glands regressed or if over 50% residual hyperplasia was evident, and the extent of hyperplasia was like or worse than that before progesterone treatment^58^, ^59^.

### Cell lines and cell culture

The human EC cell line ISK was purchased from the Chinese National Collection of Authenticated Cell Cultures. The human embryonic kidney cell line HEK293T was obtained from the ATCC and maintained in our laboratory. All cells were cultured in Dulbecco's Modified Eagle Medium (Gibco) supplemented with 10% (v/v) fetal bovine serum (FBS; Gibco), 100 U/mL penicillin G, and 100 μg/mL streptomycin (Gibco, #15140-122) at 37 °C in a humidified chamber with 5% CO_2_. MPA-resistant ISK_Res cells were generated from parental ISK cells using gradually increasing concentrations of MPA over approximately 6 months. Resistance status was examined by calculating the half-maximal inhibitory concentration (IC50) of MPA. The ISK cell line was authenticated by Shanghai Biowing Applied Biotechnology Co., Ltd. (http://www.biowing.com.cn) using short tandem repeat (STR) genetic analysis. All cells were routinely tested for Mycoplasma infection.

### Reagents and antibodies

Medroxyprogesterone acetate (S2567), MLN4924 (S7109), MG132 (S2619), BAY11-7082 (S2913), CHX (S7418), rapamycin (S1039) and CQ (S6999) were purchased from Selleck Chemicals and were dissolved in dimethylsulfoxide (DMSO, MCE, HY-Y0320) and store at -20 °C.

Antibodies specific for the following proteins were used in this study: AMBRA1 (13762-1-AP, Proteintech, dilution: 1:1000 for WB, 1:500 for IP, and 1:100 for IHC), PR (ab191138, Abcam, dilution: 1:1000 for WB, 1:500 for IP, and 1:100 for IHC), CUL4A (66038-1-Ig, Proteintech, dilution: 1:1000 for WB and 1:500 for IP), CUL4B (A12696, ABclonal, dilution: 1:1000 for WB), DDB1 (11380-1-AP, Proteintech, dilution: 1:1000 for WB and 1:500 for IP), NAE1 (A4254, ABclonal, dilution: 1:1000 for WB), RANKL (A13567, ABclonal, dilution: 1:1000 for WB), LC3B (A19665, ABclonal, dilution: 1:1000 for WB), IKKα (A2062, ABclonal, dilution: 1:1000 for WB and 1:500 for IP), HA (3724, Cell Signaling Technology, dilution: 1:2000 for WB), FLAG (14793, Cell Signaling Technology, dilution: 1:2000 for WB), MYC (2276, Cell Signaling Technology, dilution: 1:2000 for WB), His (AE104, ABclonal, dilution: 1:1000 for WB), His (AE003, ABclonal, dilution: 1:500 for IP), and β-actin (A2228, Sigma-Aldrich, dilution: 1:5000 for WB).

### Plasmids and mutagenesis

Full-length cDNA of PR (NM_000926.4) and AMBRA1 (NM_001367468.1) were amplified by PCR and cloned into vectors. Truncated AMBRA1 and PR mutants were cloned from WT plasmids. All fragments and vectors were purified from the agarose gel using a Gel Extraction Kit (Sangon, #B511139-0050) and assembled using the ClonExpress Ultra One Step Cloning Kit V2 (Vazyme, #C116) according to the manufacturer's protocols. Point mutations in the PR were generated using a Fast Site-Directed Mutagenesis Kit (TransGen Biotech, #FM111). HA-Ub, HA-Ub-K6R, HA-Ub-K11R, HA-Ub-K27R, HA-Ub-K29R, HA-Ub-K33R, HA-Ub-K48R, and HA-Ub-K63R plasmids were kindly provided by Dr. Jiaying Huang. All plasmids were verified by Sanger sequencing. Cells were transiently transfected with the indicated plasmids using jetPRIME reagent (Polyplus, #101000046) according to the manufacturer's instructions.

### Generation of stable cell lines

To overexpress AMBRA1 in ISK cells, the *AMBRA1* coding sequence (CDS) was cloned into the pCDH-puro lentiviral vector. For CRISPR/Cas9-mediated *AMBRA1* knockout, small guide RNA (sgRNA) oligonucleotides specifically targeting AMBRA1 were inserted into the lenti-guide puro vector. Lentiviruses were produced in HEK293T cells by co-transfecting the indicated lentiviral constructs with pCMV-VSV-G/pCMV-dR8.9 envelope/packaging plasmids (Polyplus, #101000046), according to the manufacturer's instructions. Forty-eight to seventy-two hours after transfection, viral supernatants were collected, cleared, and used to infect target cells for 24 h. Infected cells were then selected with 1 μg/mL puromycin for 1 week to establish stable *AMBRA1*-overexpressing cell lines. To generate AMBRA1-KO cell lines, the infected cells were selected with 1 μg/mL puromycin for 3 days and then plated at a single-cell density in 100 mm dishes, and the individual clones that emerged were picked and replated into 24-well plates. Loss of AMBRA1 expression was confirmed using immunoblotting.

Human AMBRA1 sgRNA sequences were as follows:

sgAMBRA1#1: 5'-GTGAGTAAGTAGTGTCCAAG-3',

sgAMBRA1#2: 5'-TGTTAACCCCTCTAATCAAC-3'.

### Establishment and culture of human endometrial cancer organoids

ECOs were generated and cultured as described previously^60^. Briefly, primary tumor tissues obtained from surgery were minced into small fragments and incubated in digestion buffer containing 1.0 mg/mL collagenase I (Gibco, #17100-017) and 0.5 mg/mL collagenase IV (Gibco, #17104-019) in DMEM/F12 (Gibco, #11330500BT) at 37 °C for 30 min. The cell suspension was passed through a 100-μm cell strainer (JET BIOFIL, #CSS-013-100) and centrifuged at 350×g for 5 min to collect the cells. The pellet was washed once with DMEM/F12 and resuspended in Matrigel (Organpharma, #NGH030024) at a 1:2 (v/v) ratio. Aliquots of 30 μL were plated onto the bottom of 48-well plates to form hemispherical domes. After Matrigel solidification, 160 μL ECO Culture Medium (Organpharma, #NGH020008) was added to each well. Cultures were maintained at 37 °C in a humidified incubator with 5% CO₂, and organoid growth was monitored with medium changes every 3 days. Organoids were passaged every 8-12 days or used for further experiments. For passaging, organoids were dissociated with Organoid Digestion Solution (Organpharma, #NGH030022) at 37 °C for 4 min and passaged at a split ratio of 1:2-1:3. This procedure disrupted the spherical organoids into single cells, which were then re-embedded in cold Matrigel.

To overexpress AMBRA1 in ECOs, digested organoids were resuspended in culture medium containing viral supernatant supplemented with polybrene in a 48-well plate. The samples were then centrifuged at 800×g for 1 h, followed by incubation at 37 °C for 6 h. After incubation, the solution was collected and centrifuged at 350×g for 5 min to remove the viral supernatant. Subsequently, the cells were resuspended in ice-cold Matrigel and seeded onto the bottom of 48-well plates for continuous culture.

### Cell proliferation, Annexin V staining and colony formation assay

Cell proliferation was assessed using the Cell Counting Kit-8 (CCK8; Vazyme, #A311-01). Briefly, cells were seeded into 96-well plates at a density of 2,000 cells/well and cultured under the indicated treatment conditions. Cell viability was measured at 0, 24, 48, and 72 h, according to the manufacturer's instructions.

For apoptotic analysis, cells were seeded in 6-well plates (5 × 10^5^ cells/well), treated with the indicated drugs at 37 °C for 72 h, and then harvested for staining with Annexin V (BioLegend, #640920) and 7-aminoactinomycin D (7-AAD; BioLegend, #420404) staining. Finally, the cells were analyzed using a BD Fortessa Flow cytometer (BD Biosciences, Franklin Lakes, NJ, USA) or CytoFLEX Research Flow Cytometer (Beckman Coulter), and the data were analyzed using FlowJo software.

For the clonogenic assay, cells were seeded at 1,500 cells/well in 6-well plates and treated with the indicated drugs for 10 d. Colonies were fixed with 4% paraformaldehyde at room temperature for 20 min, stained with 0.1% crystal violet for 10 min and counted.

### RNA interference

The cells were transfected with siRNA oligonucleotides (GenePharma) using Lipofectamine RNAiMAX (Invitrogen, #13778-075) according to the manufacturer's instructions. The following siRNA sequences were used:

siAMBRA1-1: 5'-GGCCUAUGGUACUAACAAAUU-3',

siAMBRA1-2: 5'-GAGUAGAACUGCCGGAUAGUU-3',

siCUL4A-1: 5'-GAACUUCCGAGACAGACCUUU-3',

siCUL4A-2: 5'-GCAGAACUGAUCGCAAAGCAUUU-3',

siCUL4B-1: 5'-GGUGCUGCUAAUGUUUAAUTT -3',

siCUL4B-2: 5'-GGCCUAGCCAAAUCUUCUATT -3',

siDDB1-1: 5'-GCUGAGUGCUUGACAUACCUUGAUA-3',

siDDB1-2: 5'-UCGCUCCAUCCUGAUGACCACCUUU-3'.

### Immunoblotting, immunoprecipitation, and ubiquitination assays

For the direct immunoblotting assay, cells (1 × 10^6^) were lysed in 100 μL of 2x sodium dodecyl sulfate (SDS) sample buffer containing radioimmunoprecipitation assay buffer, phenylmethylsulfonyl fluoride, and protease inhibitors and boiled at 100 °C for 10 min. Equal amounts of protein were separated by sodium dodecyl sulfate-polyacrylamide gel electrophoresis and electrotransferred onto polyvinylidene difluoride membranes (Millipore, Burlington, MA, USA). After blocking with 5% nonfat milk, the membranes were incubated with primary antibodies at 4 °C overnight on a rotating platform. The protein was detected using an HRP-conjugated secondary antibody, and the immunoreaction was visualized by enhanced chemiluminescence.

For immunoprecipitation, the cells were lysed with NP40 lysis buffer (Beyotime Biotechnology, #P0013F) containing a protease inhibitor cocktail and phosphatase inhibitors. Protein lysates were subjected to immunoprecipitation with anti-FLAG/HA magnetic beads (YEASEN, #20565ES03, #20566ES03) or protein A/G magnetic beads (Vazyme, #PB101-02) conjugated with a primary antibody at 4 °C for 4 h. The beads were washed with NP-40 buffer and analyzed by immunoblotting.

To detect the ubiquitination level of PR, HEK293T cells were transfected with plasmids or siRNAs, as indicated. After transfection for 36 h, the cells were treated with 20 μM MG132 for 6 h. Cells were collected and lysed in denaturing lysis buffer (50 mM Tris-HCl [pH 7.4], 150 mM NaCl, 1% NP40, and 1% SDS with protease and phosphatase inhibitors). Cell lysates were boiled for 5 min, diluted 10 times in lysis buffer without SDS, and subjected to immunoprecipitation with FLAG beads, followed by immunoblotting.

### Immunoprecipitation mass spectrum

ISK_Res cells were treated with MG132 (20 µM) for 6h. Cells were lysed and subjected to immunoprecipitation using an anti-AMBRA1 antibody or an IgG. After SDS-PAGE gel electrophoresis, the gel bands were excised and subjected to 10 mM dithiothreitol (DTT) for 40 min at 56 °C, followed by alkylation with 50 mM iodoacetamide (IAM) for 30 min in the dark at room temperature. The gel was then excised and treated with 10 ng/μL of sequencing-grade modified trypsin overnight at 37 °C for protein digestion. Digestion was terminated by adding 0.1% formic acid (FA). The supernatants were collected, and the peptides were extracted using 30% acetonitrile (ACN). The resulting peptide mixtures were dried and reconstituted in 0.1% FA for mass spectrometry analysis.

For LC-MS analysis, a nanoflow EASY-nLCTM 1200NA upgrade UHPLC system (Thermo Fisher, Germany) coupled with an Orbitrap Q Exactive^TM^ HF-X mass spectrometer (Thermo Fisher, Germany) was used. Samples were analyzed on an EASY-nLCTM 1200NA upgrade UHPLC system with a homemade C18 analytical column (75 μm × 4.5 cm, 3 μm). The mobile phase comprised solutions A (0.1% FA) and B (0.1% FA in 80% ACN). The derivatized peptides were separated in a homemade analytical column (15 cm×150 μm, 1.9 μm) using the following gradients: 5%-8% B in 2 min, 8%-44% B in 38 min, 44%-70% B in 8 min, 70%-100% B in 2 min, and 100% B for 10 min, at a flow rate of 200 nL min. High-field asymmetric waveform ion mobility spectrometry (FAIMS) was performed during data acquisition with compensation voltages of 40 V and 60 V. MS1 data were collected using an Orbitrap (60,000 resolution). Charge states between 2 and 7 were required for MS2 analysis, and a 45 s dynamic exclusion window was applied. The cycle time was set to 1 s. MS2 scans were performed using an Orbitrap with HCD fragmentation (isolation window, 1.6; 15,000 resolution; normalized collision energy (NCE), 30%).

The data were processed using the UniProt human SwissProt protein database (20436 proteins) and Protein Discoverer (version 2.4; Thermo Fisher Scientific) with Mascot (version 2.7.0; Matrix Science). The mass tolerances were 10 ppm for the precursor and fragment mass tolerance of 0.02 Da. Up to two missed cleavages were allowed. The minimum number of unique peptides used for protein identification was set at one. The search engine set cysteine carbamidomethylation as a fixed modification, N-acetylation in the proteins, and oxidation of methionine as variable modifications.

### 4D label-free quantitative proteomics analysis

To identify the binding partner of PR, ISK and ISK_Res cells were transiently transfected with the FLAG-PR plasmid, and after MPA treatment for 24 h, cells were lysed and immunoprecipitated with FLAG beads (n = 3). The immunoprecipitates were digested with sequencing-grade trypsin and desalted. The desalted peptides were separated using a Vanquish Neo UPLC system (Thermo Fisher Scientific) and subjected to MS/MS analysis using a timsTOF HT mass spectrometer (Bruker). Both precursor and fragment ions were detected using a TOF analyzer. The timsTOF HT was operated in data-independent acquisition parallel accumulation-serial fragmentation (dia-PASEF) mode. Full MS scans were acquired over a mass range of m/z 300-1500, with 12 PASEF MS/MS scans collected per cycle. MS/MS spectra were acquired over a mass range of m/z 395-1080 using an isolation window of 15 m/z. DIA data were processed using the DIA-NN search engine (version 1.8). Tandem mass spectra were searched against the *Homo sapiens* UniProt Swiss-Prot database (Homo_sapiens_9606_SP_20241202.fasta, 20,422 entries) concatenated with a reverse decoy database. Trypsin/P was specified as the proteolytic enzyme, allowing up to one missed cleavage. Methionine excision at the protein N-terminus and carbamidomethylation of cysteine residues were set as fixed modifications. The false discovery rate (FDR) was adjusted to <1%.

### Quantitative RT-PCR analysis

Total RNA was extracted using TRIzol reagent, followed by isopropanol precipitation. A total of 1 μg of RNA was reverse transcribed into cDNA using the First-Strand cDNA Synthesis Kit (TransGen, #AU341-02). Quantitative PCR was performed on an Applied Biosystems 7500 Fast Real-Time PCR system (Foster City, CA, USA) using the qPCR SuperMix (TransGen, #AQ141-02). Relative mRNA expression was calculated using the 2^-ΔΔCt^ method and normalized to β-actin expression. All reactions were performed in triplicate. The detailed sequences of the PCR primers used in this study are listed in Supplementary Table 2.

### Dual-luciferase reporter assays

Cells were transfected with the pGL4.10-RANKL promoter plasmid, firefly plasmid, or pGL4.74 [hRluc/TK] Renilla plasmid, which served as an internal control. The pGL4.10-RANKL promoter plasmids containing putative PR-binding sites (AACATAT)^9^ were co-transfected with the PR overexpression plasmid or its vector control plasmid using the jetPRIME reagent (Polyplus, #101000046) according to the manufacturer's instructions. The cells were then lysed to detect luciferase activity using a dual-luciferase reporter kit (YEASEN, #11402ES60).

### *In vivo* xenograft mouse model

All animal experiments were conducted in strict accordance with the Guidelines for the Care and Use of Laboratory Animals and Animal Facility. Four-week-old female BALB/c athymic mice were subcutaneously injected with stably transfected cells (1 × 10^7^ cells in 100 μL PBS). After tumor establishment, the mice were randomly divided into different groups and received either control or MPA treatment (i.p., 20 mg/kg body weight every other day). For combination treatment, MLN4924 (i.p., 60 mg/kg body weight every other day) was administered. Tumor formation was closely monitored after injection, and the tumor volume was measured twice a week according to the following formula: volume (mm^3^) = L (major axis) × W^2^ (minor axis)/2. The experiments were performed in an observer-blinded and randomized manner.

### H&E and immunohistochemical (IHC) staining

Tissues were fixed in 4% paraformaldehyde, dehydrated, paraffin-embedded, and cut into 5-μm-thick sections. The sections were stained with hematoxylin for 1 min to identify the cell nuclei and eosin for 30 s to identify the cytoplasm. Finally, the sheets were dehydrated and sealed.

For IHC staining, paraffin-embedded slides were deparaffinized, rehydrated, and blocked with 3% H_2_O_2_. Antigen retrieval was performed using a citrate buffer. The sections were then blocked with 10% goat serum at 37 °C for 1 h and subsequently incubated with the indicated primary antibodies at 4 °C overnight. followed by a PBS wash and then incubated with a secondary antibody for 1 h at 37 °C. Positive staining was visualized using 3,3'-diaminobenzidine (DAB) and counterstained with hematoxylin. Images of the stained sections were captured using an Aperio ScanScope slide scanner (Leica Microsystems). For quantitative analysis, three sections per sample were analyzed using the ImageJ software.

### Statistics analysis

Data from two groups were analyzed using the unpaired Student's t-test, and data from three or more groups were analyzed using analysis of variance. GraphPad Prism 10 software was used for all graphical and statistical analyses, with *p-values* of < 0.05 considered statistically significant. The experimental results are expressed as the mean ± standard deviation.

## Supplementary Material

Supplementary figures and tables.

## Data Availability

The mass spectrometry proteomic data generated in this study were deposited in the ProteomeXchange Consortium via the iProX partner repository under accession code PXD072640 and PXD072904. The remaining data are available in the article and supplementary files. The source data were provided in this study.

## Abbreviations

EC: Endometrial cancer
MPA: Medroxyprogesterone acetate
PR: progesterone receptor
LBD: ligand-binding domain
PRB: progesterone receptor B
AMBRA1: autophagy/Beclin-1 regulator 1
CRLs: Cullin-RING ligases
CRL4: CUL4-RING E3 ubiquitin ligase
IHC: immunohistochemistry
NSMP: No specific molecular profile
ECOs: Endometrial cancer organoids
Co-IP: co-immunoprecipitation
CHX: cycloheximide
DCAF: DDB1- and CUL4-associated factor

## References

[1] Crosbie EJ, Kitson SJ, McAlpine JN, Mukhopadhyay A, Powell ME, Singh N (2022). Endometrial cancer. Lancet.

[2] Sung H, Ferlay J, Siegel RL, Laversanne M, Soerjomataram I, Jemal A (2021). Global Cancer Statistics 2020: GLOBOCAN Estimates of Incidence and Mortality Worldwide for 36 Cancers in 185 Countries. CA Cancer J Clin.

[3] Siegel RL, Giaquinto AN, Jemal A (2024). Cancer statistics, 2024. CA Cancer J Clin.

[4] Kovacevic N (2021). Surgical treatment and fertility perservation in endometrial cancer. Radiol Oncol.

[5] van Weelden WJ, Lalisang RI, Bulten J, Lindemann K, van Beekhuizen HJ, Trum H (2021). Impact of hormonal biomarkers on response to hormonal therapy in advanced and recurrent endometrial cancer. Am J Obstet Gynecol.

[6] Corr BR, Erickson BK, Barber EL, Fisher CM, Slomovitz B (2025). Advances in the management of endometrial cancer. Bmj.

[7] Duska LR, Filiaci VL, Walker JL, Holman LL, Hill EK, Moore RG (2021). A Surgical Window Trial Evaluating Medroxyprogesterone Acetate with or without Entinostat in Patients with Endometrial Cancer and Validation of Biomarkers of Cellular Response. Clin Cancer Res.

[8] Zhang C, Sheng Y, Sun X, Wang Y (2023). New insights for gynecological cancer therapies: from molecular mechanisms and clinical evidence to future directions. Cancer Metastasis Rev.

[9] Yang L, Fan Q, Wang J, Yang X, Yuan J, Li Y (2023). TRPS1 regulates the opposite effect of progesterone via RANKL in endometrial carcinoma and breast carcinoma. Cell Death Discov.

[10] Kim JJ, Chapman-Davis E (2010). Role of progesterone in endometrial cancer. Semin Reprod Med.

[11] Li J, Qin Z, Li Y, Huang B, Xiao Q, Chen P (2024). Phosphorylation of IDH1 Facilitates Progestin Resistance in Endometrial Cancer. Adv Sci (Weinh).

[12] Mani S, Portillo W (2010). Activation of progestin receptors in female reproductive behavior: Interactions with neurotransmitters. Front Neuroendocrinol.

[13] Suzuki Y, Ferris JS, Chen L, Dioun S, Usseglio J, Matsuo K (2024). Fertility-preserving treatment for stage IA endometrial cancer: a systematic review and meta-analysis. Am J Obstet Gynecol.

[14] Zhou S, Xu Z, Yang B, Guan J, Shan W, Shi Y (2021). Characteristics of progestin-insensitive early stage endometrial cancer and atypical hyperplasia patients receiving second-line fertility-sparing treatment. J Gynecol Oncol.

[15] Dai D, Wolf DM, Litman ES, White MJ, Leslie KK (2002). Progesterone inhibits human endometrial cancer cell growth and invasiveness: down-regulation of cellular adhesion molecules through progesterone B receptors. Cancer Res.

[16] Lee II, Maniar K Lydon JP, Kim JJ (2016). Akt regulates progesterone receptor B-dependent transcription and angiogenesis in endometrial cancer cells. Oncogene.

[17] Hu Z, Wu Z, Liu W, Ning Y, Liu J, Ding W (2024). Proteogenomic insights into early-onset endometrioid endometrial carcinoma: predictors for fertility-sparing therapy response. Nat Genet.

[18] Zaino RJ, Brady WE, Todd W, Leslie K, Fischer EG, Horowitz NS (2014). Histologic effects of medroxyprogesterone acetate on endometrioid endometrial adenocarcinoma: a Gynecologic Oncology Group study. Int J Gynecol Pathol.

[19] Yang S, Xiao X, Jia Y, Liu X, Zhang Y, Wang X (2014). Epigenetic modification restores functional PR expression in endometrial cancer cells. Curr Pharm Des.

[20] Xiong Y, Dowdy SC, Gonzalez Bosquet J, Zhao Y, Eberhardt NL, Podratz KC (2005). Epigenetic-mediated upregulation of progesterone receptor B gene in endometrial cancer cell lines. Gynecol Oncol.

[21] Sasaki M, Dharia A, Oh BR, Tanaka Y, Fujimoto S, Dahiya R (2001). Progesterone receptor B gene inactivation and CpG hypermethylation in human uterine endometrial cancer. Cancer Res.

[22] Cui Y, Wu H, Yang L, Huang T, Li J, Gong X (2021). Chlorpromazine Sensitizes Progestin-Resistant Endometrial Cancer Cells to MPA by Upregulating PRB. Front Oncol.

[23] Cheng Y, Xie L, Xu Z, Hao M, Yang B, Shan W (2022). NrCAM secreted by endometrial stromal cells enhances the progestin sensitivity of endometrial cancer cells through epigenetic modulation of PRB. Cancer Gene Ther.

[24] Xu W, Hua Z, Wang Y, Tang W, Ou W, Liu F (2024). AMBRA1 promotes intestinal inflammation by antagonizing PP4R1/PP4c mediated IKK dephosphorylation in an autophagy-independent manner. Cell Death Differ.

[25] Maiani E, Milletti G, Cecconi F (2021). The pro-autophagic protein AMBRA1 coordinates cell cycle progression by regulating CCND (cyclin D) stability. Autophagy.

[26] La Barbera L, Vedele F, Nobili A, D'Amelio M, Krashia P (2019). Neurodevelopmental Disorders: Functional Role of Ambra1 in Autism and Schizophrenia. Molecular neurobiology.

[27] Fimia GM, Di Bartolomeo S, Piacentini M, Cecconi F (2011). Unleashing the Ambra1-Beclin 1 complex from dynein chains: Ulk1 sets Ambra1 free to induce autophagy. Autophagy.

[28] Qiu X, Li N, Yang Q, Wu S, Li X, Pan X (2023). The potent BECN2-ATG14 coiled-coil interaction is selectively critical for endolysosomal degradation of GPRASP1/GASP1-associated GPCRs. Autophagy.

[29] Strappazzon F, Di Rita A, Peschiaroli A, Leoncini PP, Locatelli F, Melino G (2020). HUWE1 controls MCL1 stability to unleash AMBRA1-induced mitophagy. Cell Death Differ.

[30] Di Rienzo M, Romagnoli A, Ciccosanti F, Refolo G, Consalvi V, Arena G (2022). AMBRA1 regulates mitophagy by interacting with ATAD3A and promoting PINK1 stability. Autophagy.

[31] Di Leo L, De Zio D (2021). AMBRA1 has an impact on melanoma development beyond autophagy. Autophagy.

[32] Di Leo L, Bodemeyer V, Bosisio FM, Claps G, Carretta M, Rizza S (2021). Loss of Ambra1 promotes melanoma growth and invasion. Nat Commun.

[33] Liu J, Yuan B, Cao J, Luo H, Gu S, Zhang M (2021). AMBRA1 Promotes TGFβ Signaling via Nonproteolytic Polyubiquitylation of Smad4. Cancer Res.

[34] Fu DJ, Wang T (2023). Targeting NEDD8-activating enzyme for cancer therapy: developments, clinical trials, challenges and future research directions. J Hematol Oncol.

[35] Wu K, Huynh KQ, Lu I, Moustakim M, Miao H, Yu C (2021). Inhibitors of cullin-RING E3 ubiquitin ligase 4 with antitumor potential. Proc Natl Acad Sci U S A.

[36] Ma X, Zhao T, Yan H, Guo K, Liu Z, Wei L (2021). Fatostatin reverses progesterone resistance by inhibiting the SREBP1-NF-κB pathway in endometrial carcinoma. Cell Death Dis.

[37] Simoneschi D, Rona G, Zhou N, Jeong YT, Jiang S, Milletti G (2021). CRL4(AMBRA1) is a master regulator of D-type cyclins. Nature.

[38] Maiani E, Milletti G, Nazio F, Holdgaard SG, Bartkova J, Rizza S (2021). AMBRA1 regulates cyclin D to guard S-phase entry and genomic integrity. Nature.

[39] Chaikovsky AC, Li C, Jeng EE, Loebell S, Lee MC, Murray CW (2021). The AMBRA1 E3 ligase adaptor regulates the stability of cyclin D. Nature.

[40] Zakhour M, Cohen JG, Gibson A, Walts AE, Karimian B, Baltayan A (2017). Abnormal mismatch repair and other clinicopathologic predictors of poor response to progestin treatment in young women with endometrial complex atypical hyperplasia and well-differentiated endometrial adenocarcinoma: a consecutive case series. Bjog.

[41] Di Rita A, Peschiaroli A, P DA, Strobbe D, Hu Z, Gruber J (2018). HUWE1 E3 ligase promotes PINK1/PARKIN-independent mitophagy by regulating AMBRA1 activation via IKKα. Nat Commun.

[42] Nazio F, Strappazzon F, Antonioli M, Bielli P, Cianfanelli V, Bordi M (2013). mTOR inhibits autophagy by controlling ULK1 ubiquitylation, self-association and function through AMBRA1 and TRAF6. Nat Cell Biol.

[43] Tang Y, Qiu J, Tang Z, Li G, Gu M, Wang Y (2022). P38α MAPK is a gatekeeper of uterine progesterone responsiveness at peri-implantation via Ube3c-mediated PGR degradation. Proc Natl Acad Sci U S A.

[44] Huang P, Deng W, Bao H, Lin Z, Liu M, Wu J (2022). SOX4 facilitates PGR protein stability and FOXO1 expression conducive for human endometrial decidualization. Elife.

[45] Zhang PJ, Zhao J, Li HY, Man JH, He K, Zhou T (2007). CUE domain containing 2 regulates degradation of progesterone receptor by ubiquitin-proteasome. Embo j.

[46] Dere E, Dahm L, Lu D, Hammerschmidt K, Ju A, Tantra M (2014). Heterozygous ambra1 deficiency in mice: a genetic trait with autism-like behavior restricted to the female gender. Front Behav Neurosci.

[47] Heinrich A, Nees F, Lourdusamy A, Tzschoppe J, Meier S, Vollstädt-Klein S (2013). From gene to brain to behavior: schizophrenia-associated variation in AMBRA1 alters impulsivity-related traits. Eur J Neurosci.

[48] Cianfanelli V, De Zio D, Di Bartolomeo S, Nazio F, Strappazzon F, Cecconi F (2015). Ambra1 at a glance. J Cell Sci.

[49] Sun WL, He LY, Liang L, Liu SY, Luo J, Lv ML (2022). Ambra1 regulates apoptosis and chemosensitivity in breast cancer cells through the Akt-FoxO1-Bim pathway. Apoptosis.

[50] Liu J, Chen Z, Guo J, Wang L, Liu X (2019). Ambra1 induces autophagy and desensitizes human prostate cancer cells to cisplatin. Biosci Rep.

[51] Falasca L, Torino F, Marconi M, Costantini M, Pompeo V, Sentinelli S (2015). AMBRA1 and SQSTM1 expression pattern in prostate cancer. Apoptosis.

[52] Ko YH, Cho YS, Won HS, Jeon EK, An HJ, Hong SU (2013). Prognostic significance of autophagy-related protein expression in resected pancreatic ductal adenocarcinoma. Pancreas.

[53] Short NJ, Muftuoglu M, Ong F, Nasr L, Macaron W, Montalban-Bravo G (2023). A phase 1/2 study of azacitidine, venetoclax and pevonedistat in newly diagnosed secondary AML and in MDS or CMML after failure of hypomethylating agents. J Hematol Oncol.

[54] Swords RT, Erba HP, DeAngelo DJ, Bixby DL, Altman JK, Maris M (2015). Pevonedistat (MLN4924), a First-in-Class NEDD8-activating enzyme inhibitor, in patients with acute myeloid leukaemia and myelodysplastic syndromes: a phase 1 study. Br J Haematol.

[55] Qin A, Wells L, Malhotra B, Gadgeel S, Schneider BJ, Ramnath N (2024). A Phase II Trial of Pevonedistat and Docetaxel in Patients With Previously Treated Advanced Non-Small-Cell Lung Cancer. Clin Lung Cancer.

[56] Zhou X, Friedlander S, Kupperman E, Sedarati F, Kuroda S, Hua Z (2021). Asia-inclusive global development of pevonedistat: Clinical pharmacology and translational research enabling a phase 3 multiregional clinical trial. Clin Transl Sci.

[57] Dong Y, Siegwart DJ, Anderson DG (2019). Strategies, design, and chemistry in siRNA delivery systems. Adv Drug Deliv Rev.

[58] Wang S, Pudney J, Song J, Mor G, Schwartz PE, Zheng W (2003). Mechanisms involved in the evolution of progestin resistance in human endometrial hyperplasia-precursor of endometrial cancer. Gynecol Oncol.

[59] Wang Y, Yu M, Yang JX, Cao DY, Yuan Z, Zhou HM (2019). Prolonged conservative treatment in patients with recurrent endometrial cancer after primary fertility-sparing therapy: 15-year experience. Int J Clin Oncol.

[60] Zhang C, Lu X, Ni T, Wang Q, Gao X, Sun X (2024). Developing patient-derived organoids to demonstrate JX24120 inhibits SAMe synthesis in endometrial cancer by targeting MAT2B. Pharmacol Res.

