# Supplementary material for: CRL4AMBRA1-mediated progesterone receptor degradation drives progestin resistance and represents a therapeutic vulnerability in endometrial cancer
Source: Int J Biol Sci. 2026 May 1;22(9):4976–96. doi: 10.7150/ijbs.133072 (PMC13182556; doi:10.7150/ijbs.133072)
Supplement: Supplementary file 1 — Supplementary figures and tables. [file ijbsv22p4976s1.pdf]

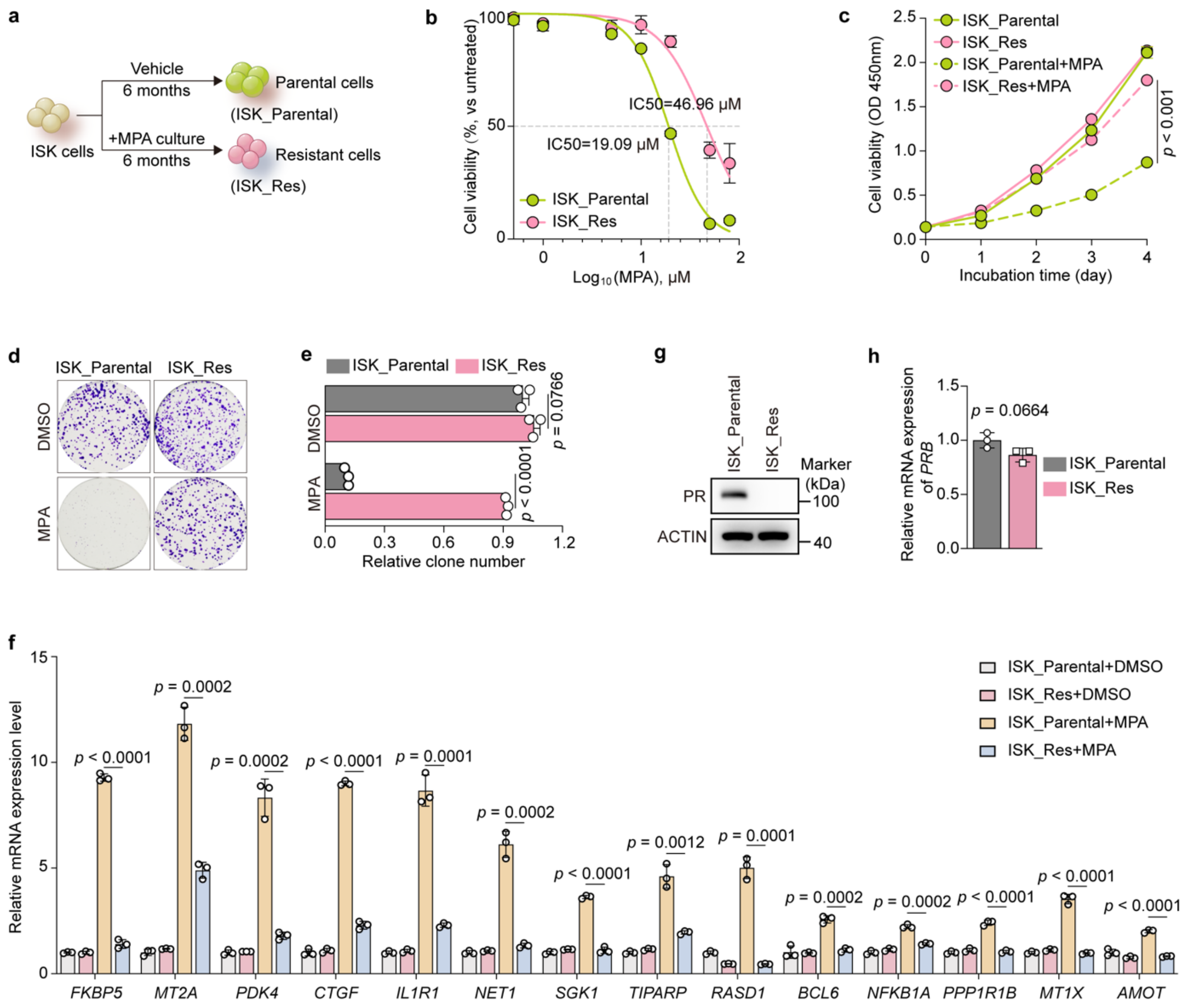

Supplementary Fig. 1. Establishment and validation of MPA-resistant EC cells.

a Illustration of the establishment of the MPA-resistant subline from ISK cells, an MPA-sensitive EC cell line. The concentration of MPA was gradually increased by 2.5 µM every 4 weeks. After 6 months of continuous treatment, the cells became resistant to MPA (ISK_Res) compared to the corresponding control cells, ISK_Parental.

b Dose-response curves for MPA in resistant (ISK_Res) and corresponding parental (ISK_Parental) cells. The cells were cultured with various concentrations of MPA for 3 days (n = four biological replicates per group).

c Cell Counting Kit 8 (CCK8) assays were performed on ISK_Res and ISK_Parental cells in response to 20 µM MPA treatment for 72 h (n = 4 biological replicates per group). OD, optical

density (n = three biological replicates per group).

d Cell growth assay by crystal violet staining of ISK_Res and ISK_Parental cells in response to 20 μM MPA for 10 days.

e Quantitative analysis of the clone numbers in (**d**) (n = three biological replicates per group).

f ISK_Res and ISK_Parental cells were treated with 20 μM MPA for 24 h, and the mRNA expression of the indicated genes was detected by qRT-PCR (n = three biological replicates per group).

g Immunoblotting analysis of PR expression levels in ISK_Res and ISK_Parental cells.

h qRT-PCR analysis of *PRB* mRNA expression levels in ISK_Res and ISK_Parental cells. (n = three biological replicates per group).

Statistical analysis in **c** was performed using a two-way ANOVA with Šídák's correction. Data in **e**, **f**, and **h**were analyzed using an unpaired two-tailed Student's t-test. All results are presented as the mean ± SD.

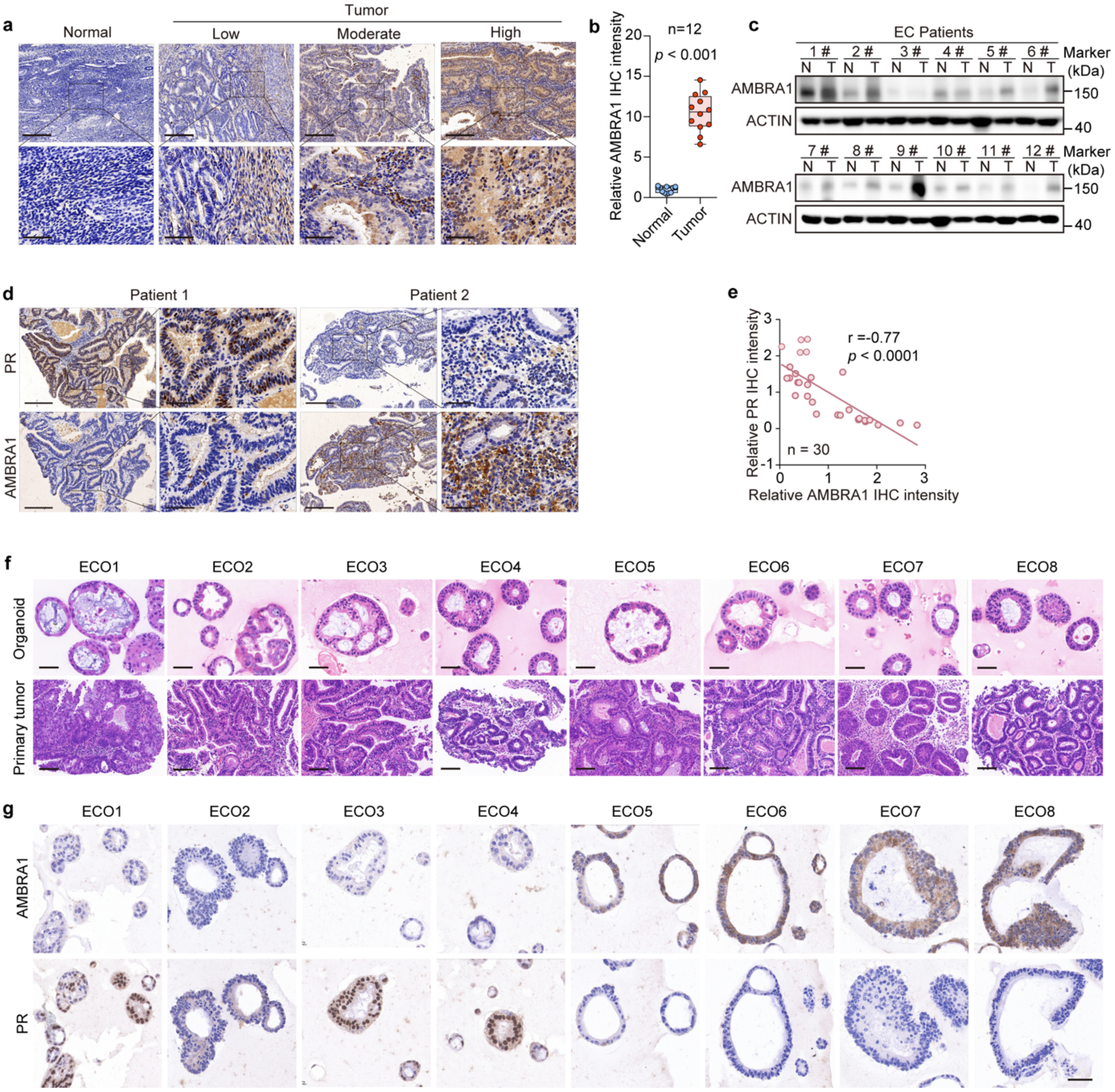

Supplementary Fig. 2. AMBRA1 is upregulated and negatively correlates with PR in patients with EC.

a IHC staining of AMBRA1 in tissues from patients with normal endometrium (n = 12) and EC (n = 12). Representative images are shown. Scale bars: upper panel, 200 μm; lower panel, 50 μm.

b Quantitative analysis of AMBRA1 IHC staining intensity in (**a**).

c Immunoblotting was used to examine AMBRA1 expression in tumor (T) and paired adjacent normal (N) endometrial tissues from patients with EC (n = 12).

d IHC staining of AMBRA1 and PR in tumor specimens from patients with EC (n=30). Representative images are shown. Scale bars: left panels, 200 μm; right panels, 50 μm.

e IHC intensities of AMBRA1 and PR (**d**) were quantified. Spearman's correlation analysis was performed to determine the correlation between AMBRA1 and PR expression in EC.

f H&E staining of the indicated ECOs and their primary tissues. Representative images are shown. Scale bars: upper panels, 50 μm; lower panels, 100 μm.

g Representative images of IHC staining for AMBRA1 and PR in the indicated ECOs. Scale bars: 50 μm.

Data in **b** were analyzed using an unpaired two-tailed Student's t-test, and the results are presented as the mean ± SD.

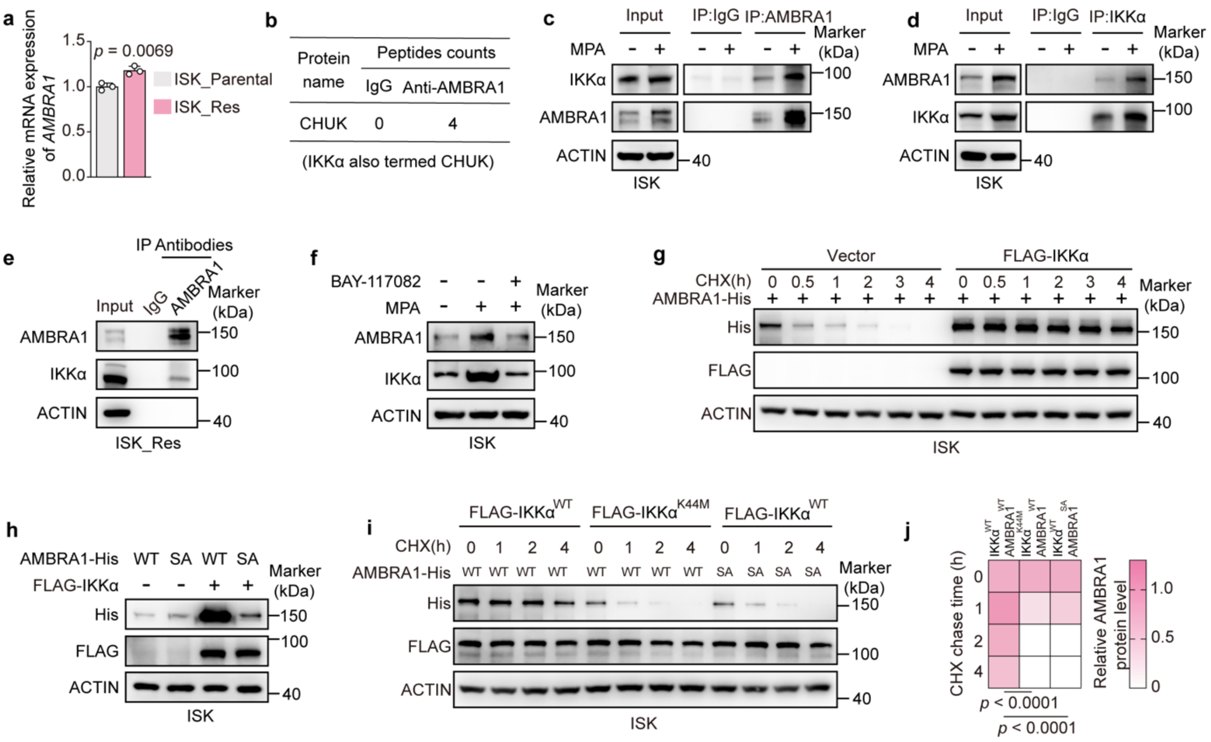

Supplementary Fig. 3. The IKKα kinase phosphorylates AMBRA1 at S1043 to stabilize the AMBRA1 protein.

a qRT-PCR analysis of *AMBRA1* mRNA expression levels in ISK_Res and ISK_Parental cells.

(n = three biological replicates per group).

b Table showing the interaction between IKKα and AMBRA1 in ISK_Res cells.

c, **d** Reciprocal endogenous Co-IP assays of the interaction between AMBRA1 and IKKα in ISK cells upon MPA treatment (20 μM, 48 h) and immunoprecipitants enriched with anti-AMBRA1 (**c**) or anti-IKKα (**d**) antibodies. IgG served as the negative control.

e Endogenous Co-IP assay of the interaction between AMBRA1 and IKKα in ISK_Res cells, and immunoprecipitant was enriched using anti-AMBRA1 antibody, with IgG serving as a negative control.

f Immunoblotting analysis of AMBRA1 and IKKα expression in ISK cells treated with MPA (20 μM, 48 h) alone or in combination with BAY11-7082 (30 μM, 24h).

g AMBRA1-His was co-transfected with the vector or FLAG-IKKα plasmid in ISK cells and treated with or without cycloheximide (CHX, 50 μg/mL) for the indicated time. Immunoblotting for AMBRA1-His and FLAG-IKKα was performed.

h ISK cells were transiently co-transfected with FLAG-IKKα and His-tagged $AMBRA1^{WT}$ or His-tagged $AMBRA1^{S1043A}$. Following a 36-hour incubation, the cells were harvested for immunoblotting analysis using anti-His and anti-FLAG antibodies.

i His-tagged $AMBRA1^{WT}$ or His-tagged $AMBRA1^{S1043A}$ was co-transfected with FLAG-$IKKα^{WT}$ or FLAG-$IKKα^{K44M}$ plasmids in ISK cells and treated with or without cycloheximide (CHX, 50 μg/mL) for the indicated time. Immunoblotting for AMBRA1-His and FLAG-IKKα was performed.

j Quantification of AMBRA1-His protein levels based on band intensity in **i** is shown.

Data in **a** were analyzed using an unpaired two-tailed Student's t-test. Statistical analysis of **j**

was performed using a two-way ANOVA with Šídák's correction. All results are presented as the mean ± SD.

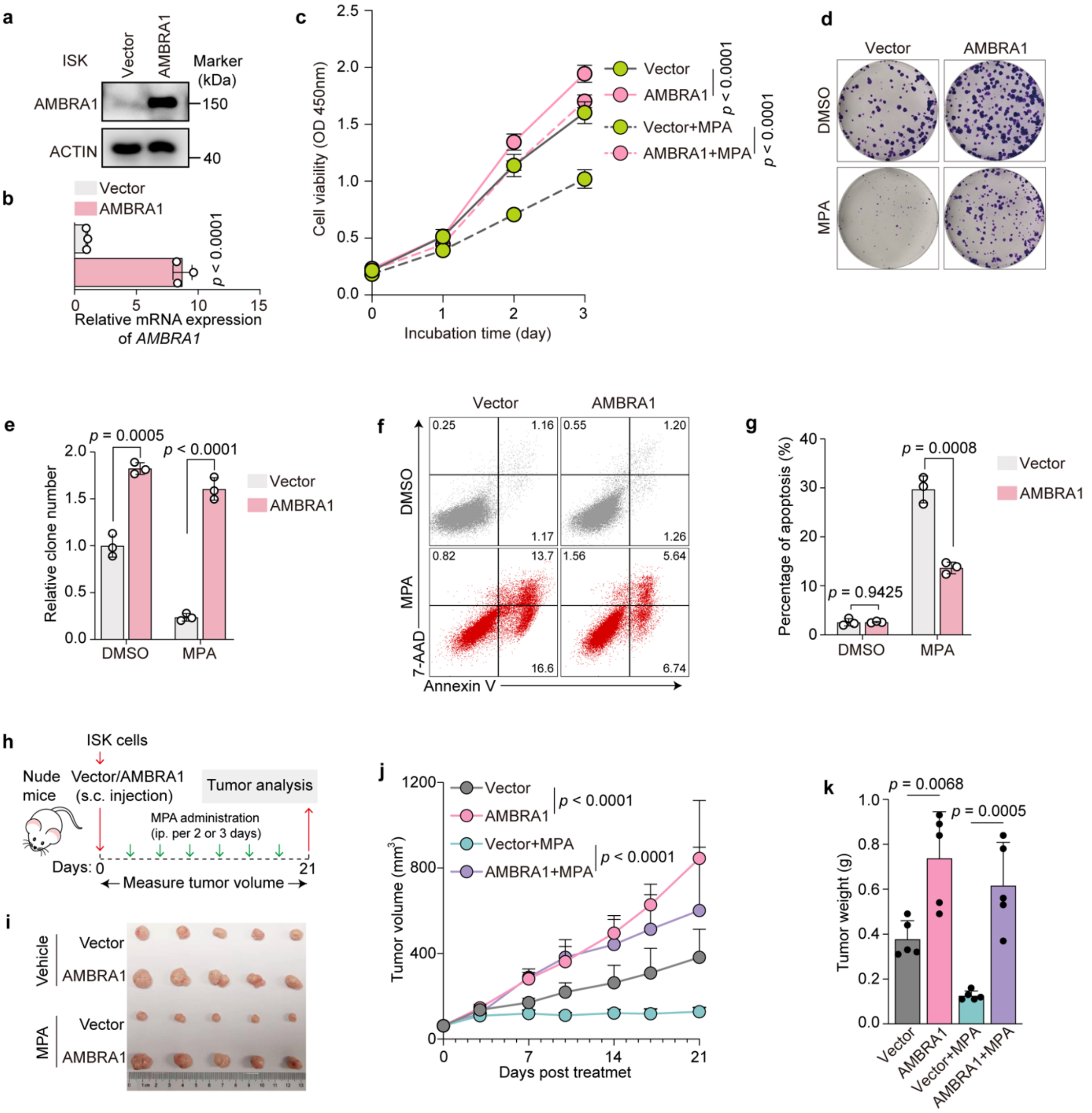

Supplementary Fig. 4. AMBRA1 overexpression promotes MPA resistance in EC cells.

a Immunoblotting confirmed the efficiency of *AMBRA1* overexpression in ISK cells.

b Efficiency of *AMBRA1* overexpression in ISK cells was examined using qRT-PCR (n = three biological replicates per group).

c CCK8 assays were performed on ISK cells with or without *AMBRA1* overexpression in

response to 20 μM MPA treatment for 72 h (n = four biological replicates per group). OD, optical density.

d Cell growth assay by crystal violet staining of ISK cells with or without *AMBRA1* overexpression in response to 20 μM MPA for 10 days.

e Quantitative analysis of the clone numbers in (**d**). (n = three biological replicates per group).

f Vector- and AMBRA1-overexpressing cells were treated with 30 μM MPA or DMSO for 72 h. Apoptosis was detected by flow cytometry after staining with Annexin V and 7-AAD.

g Percentage of apoptotic cells in (**f**) (n = three biological replicates per group).

h Diagram depicting the *in vivo* growth of tumor xenografts by subcutaneous inoculation of ISK cells generated in (**a**).

i Xenograft tumors derived from ISK cells with or without *AMBRA1* overexpression in response to MPA treatment.

j, **k** Monitored tumor volume (**j**) and weight (**k**) are shown (n = 5).

Data in **b**, **e**, **g**, and **k** were analyzed using an unpaired two-tailed Student's t-test. Statistical analyses in **c** and **j** were performed using two-way ANOVA with Šídák's correction. All results are presented as the mean ± SD.

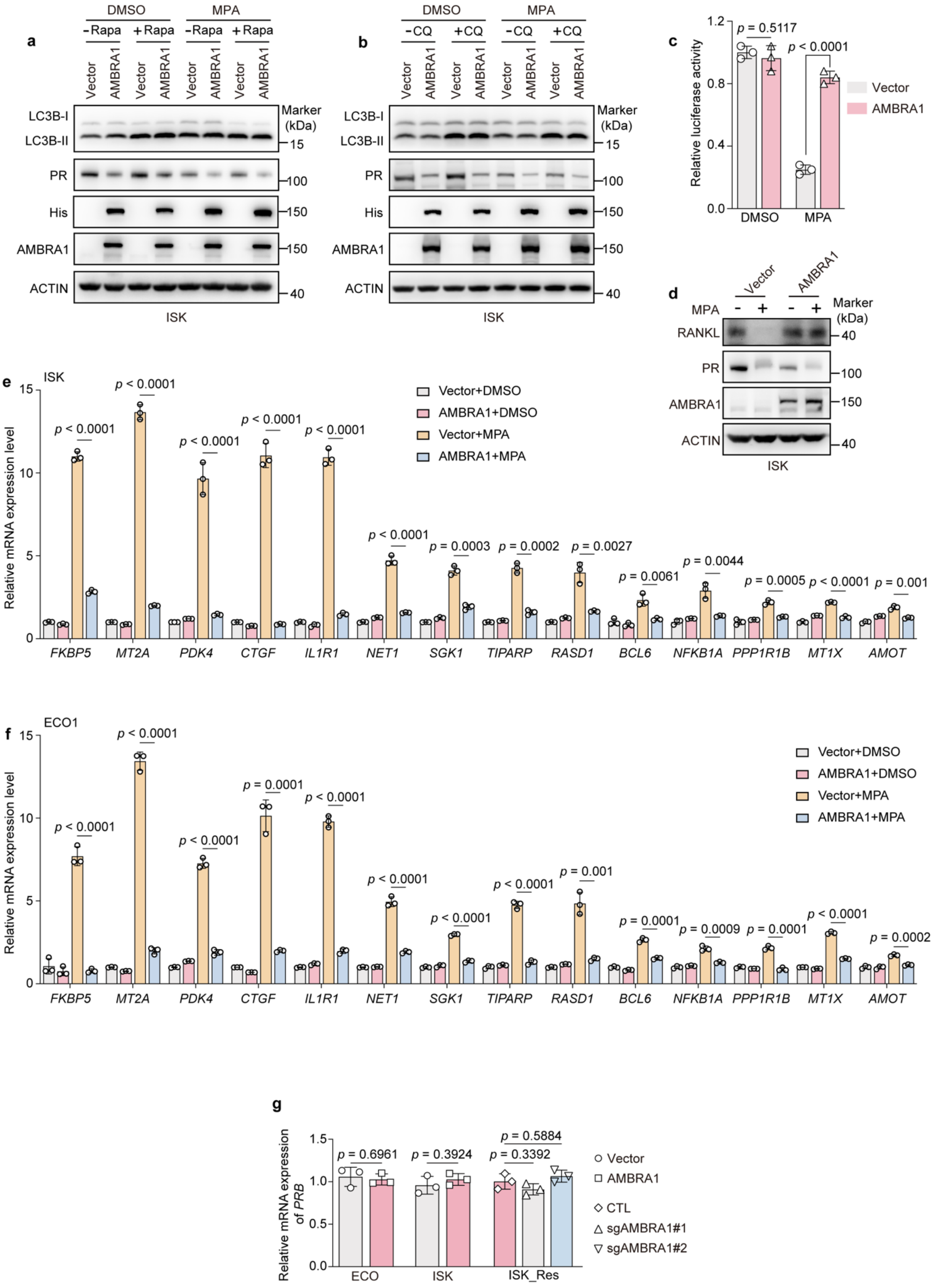

Supplementary Fig. 5. AMBRA1 inhibits MPA-induced PRB-responsive gene expression in EC cells.

a, **b** AMBRA1-overexpressing and control ISK cells were treated with rapamycin (200 nM) for 12 h (**a**) or with chloroquine (20 μM) for 6 h (**b**). Immunoblotting assays were performed to measure autophagic flux by LC3B conversion (LC3B-I to LC3B-II, which occurs during autophagosome formation) and the levels of the indicated proteins.

c ISK cells were transfected with expression plasmids for AMBRA1, RANKL-luc, and Renilla-luc, and then treated with MPA (20 μM for 24 h). Relative luciferase activity was measured (n = three biological replicates per group).

d ISK cells with or without *AMBRA1*-overexpression were treated with MPA (20μM, 24 h), and immunoblotting was used to examine the expression of the indicated proteins.

e ISK cells with or without *AMBRA1* overexpression were treated with 20 μM MPA for 24 h, and the mRNA expression of the indicated genes was detected by qRT-PCR (n = three biological replicates per group).

f ECO1 cells with or without *AMBRA1* overexpression were treated with 20 μM MPA for 24 h, and the mRNA expression of the indicated genes was detected by qRT-PCR (n = three biological replicates per group).

g qRT-PCR analysis of *PRB* mRNA expression levels in ISK cells and ECO1 with or without AMBRA1 overexpression and ISK_Res cells with or without AMBRA1 knockout (n = three biological replicates per group).

Data in **c**, **e**, **f**, and **g** (left and middle) were analyzed using an unpaired two-tailed Student's t-test. Statistical analysis in **g** (right) was performed using one-way ANOVA with Šídák's correction. All results are presented as the mean ± SD.

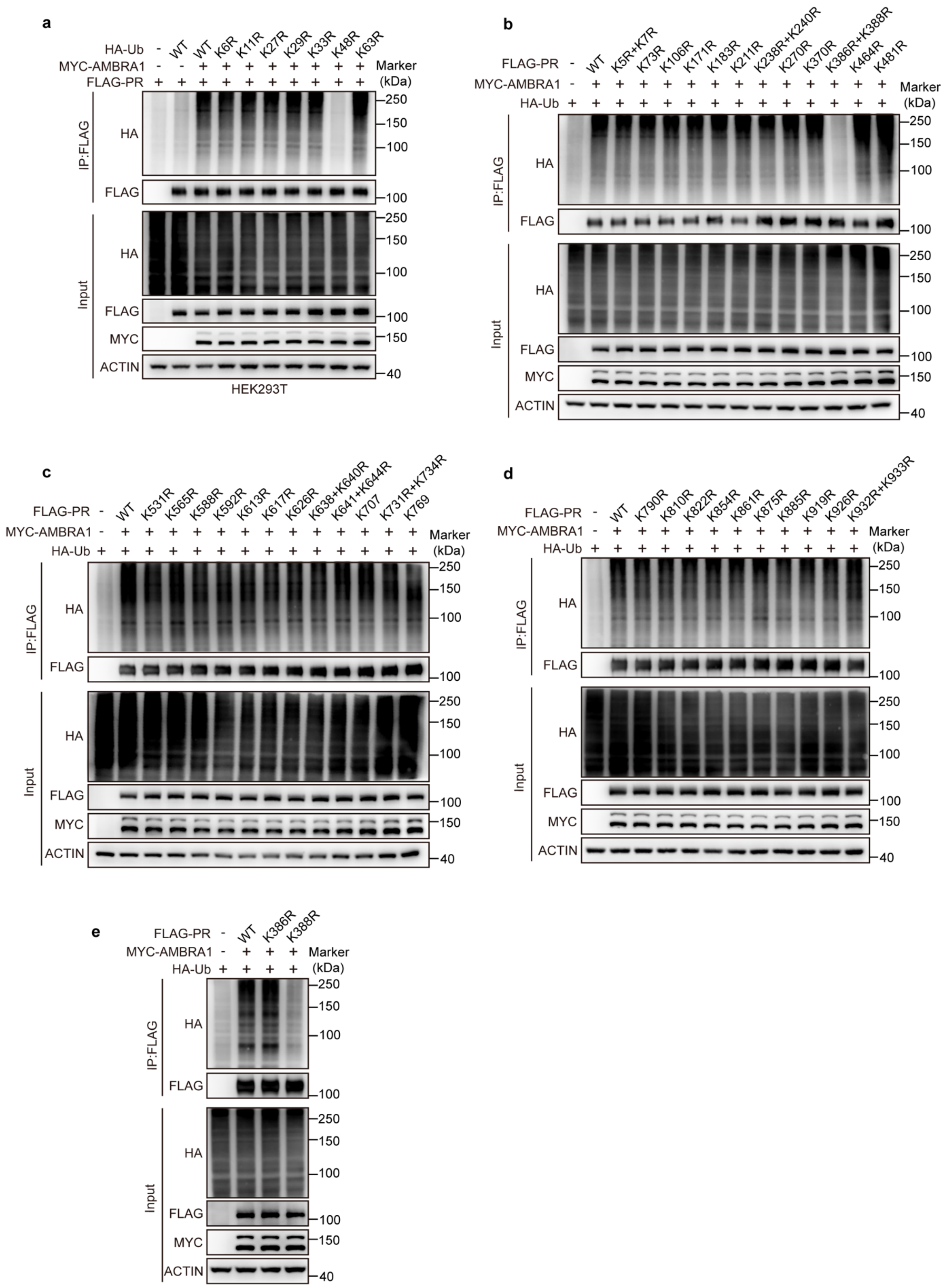

Supplementary Fig. 6. Characteristics of AMBRA1-mediated PR ubiquitylation.

a HEK293T cells were transiently transfected with plasmids expressing Myc-tagged AMBRA1,

FLAG-tagged PR, and HA-tagged wild-type (WT) Ub or Ub mutants for 36 h. After treatment with MG132 (20 µM, 6 h), polyubiquitination of PR was examined by western blotting after immunoprecipitation with FLAG Beads.

b-**e** HEK293T cells were transiently transfected with plasmids expressing Myc-tagged AMBRA1, HA-tagged Ub, FLAG-tagged wild-type PR, or 34 mutants, as indicated, for 36 h. After treatment with MG132 (20 µM, 6 h), polyubiquitination of PR was examined by immunoblotting after immunoprecipitation with FLAG Beads.

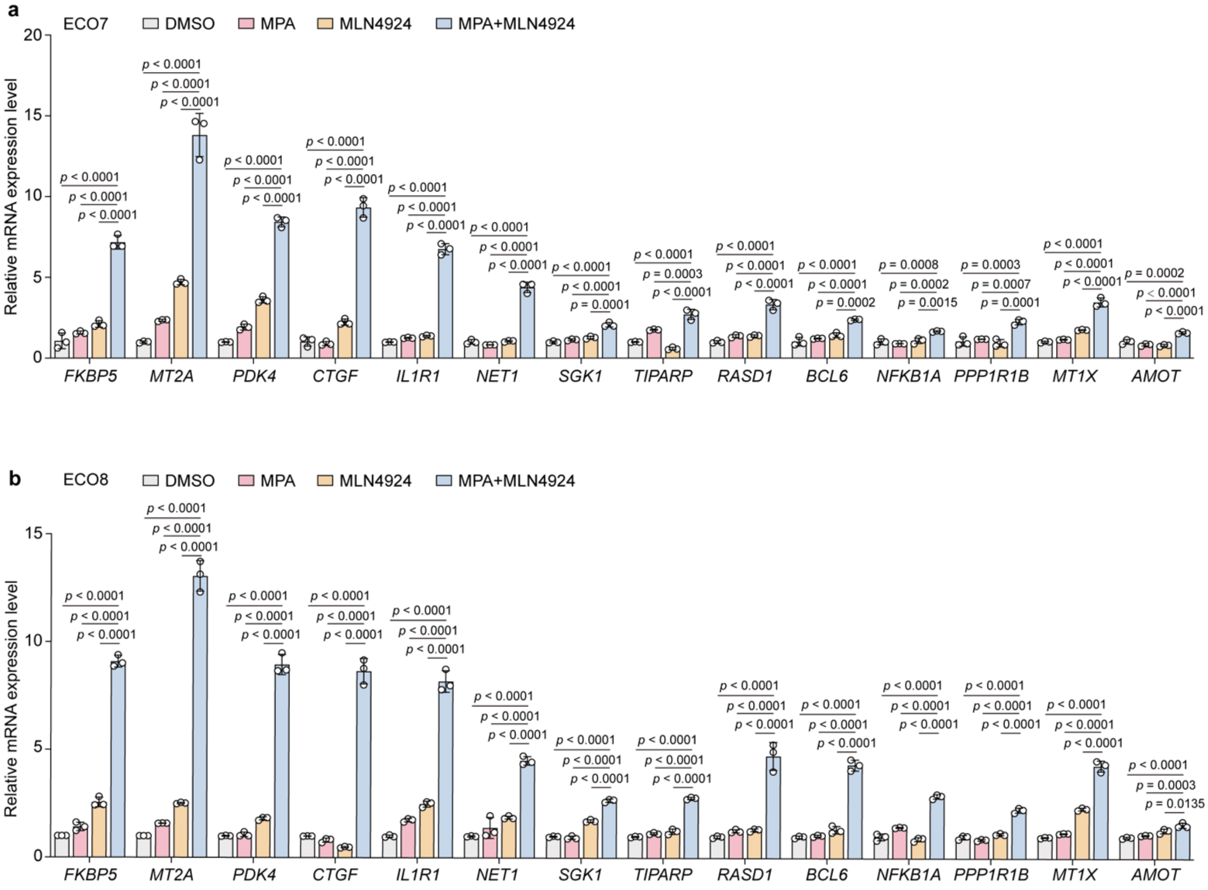

Supplementary Fig. 7. MLN4924 promotes the expression of PRB-responsive genes in MPA-resistant ECOs.

a, **b** ECO7 and ECO8 cells were treated with MPA (20 µM, 24h), MLN4924 (0.1µM, 8h) or a combination of both, and the mRNA expression of the indicated genes was detected by qRT-

PCR (n = three per group). Statistical analysis was performed using one-way ANOVA with Šídák's correction. All results are presented as the mean ± SD.

Supplementary Table 1: Differential expression of CRL components in MPA-resistant versus MPA-sensitive EC (data from GSE121367)

Gene	Log2(fold change)	p value
DCAF10	1.619474744	5.28E-06
DTL	1.230598667	4.86E-07
WDR70	1.157791	1.58E-05
CRBN	1.096734611	9.63E-07
SKP2	0.931391333	7.40E-05
AMBRA1	0.924216333	7.20E-07
RBBP7	0.838105667	0.00012774
HBXIP	0.664403667	4.65E-06
DDB2	0.586345333	3.19E-05
FBXW5	0.573674667	0.00224314
RFWD2	0.539574	0.00072151
DCAF12L1	0.448593672	0.31528394
VPRBP	0.3891158	0.00367422
FBXO44	0.350358533	0.00372905
CUL4B	0.279569167	0.00697829
CUL4A	0.2116945	0.00432412
RNF7	0.183532333	0.01249301
PHIP	0.177582667	0.01497135
AHR	0.131246333	0.13450857
BRWD1	0.033371833	0.56196437
HOXB4	-0.021356722	0.83987329
DCAF15	-0.027274533	0.8802203
GNB3	-0.056288033	0.14675784
GNB2	-0.165343167	0.21969644
DCAF17	-0.323196444	0.00044696
DCAF6	-0.429858167	0.00385684
DCAF5	-0.504396067	0.00011626
DCAF5	-0.504396067	0.00011626
CUL9	-0.543686333	0.00268879
CUL1	-0.549683667	0.00572025
DCAF16	-0.572818333	1.06E-05
DCAF7	-0.600717167	3.82E-05
DCAF7	-0.600717167	3.82E-05
DCAF8L2	-0.674420444	0.0189549
RBX1	-0.697899667	1.69E-05
CUL2	-0.714498333	1.61E-06
WDTC1	-0.7952925	0.00073905
CUL3	-0.837648333	1.02E-05
STRAP	-0.838196	2.67E-05
DCAF12L2	-0.8691445	0.00022563
DCAF13	-1.305088667	3.19E-05
DCAF11	-1.5251323	4.97E-06
COPS8	-1.675398	1.36E-05
DCAF8L1	-1.758830633	0.00098008
DCAF4	-1.790334333	6.34E-08

Supplementary Table 2: Primers for qRT-PCR in this study.

Primers	Sequences
AMBRA1-F	AACCCTCCACTGCGAGTTGA
AMBRA1-R	TCTACCTGTTCCGTGGTTCTCC
PRB-F	TGCCCAGCATGTCGCCTTAG
PRB-R	CTGGCTTAG GGCTTGGCTTTC
FKBP5-F	GCGTCCCAGAGGGGGAA
FKBP5-R	CTGGGGATTGTCGCTTCGTA
MT2A-F	GAGTGCAAATGCACTTCGTGCAA
MT2A-R	GCGTTCTTTACATCTGGGAGCG
PDK4-F	AGGTGGAGCATTTCTCGCGCTA
PDK4-R	GAATGTTGGCGAGTCTCACAGG
CTGF-F	CTTGCGAAGCTGACCTGGAAGA
CTGF-R	CCGTCGGTACATACTCCACAGA
IL1R1-F	GTGCTTTGGTACAGGGATTCCTG
IL1R1-R	CACAGTCAGAGGTAGACCCTTC
NET1-F	ATCGAAGCGAGCAAAGTGCTGC
NET1-R	CCTGGTAAGAGTGCCGTTCGTT
SGK1-F	CATATTATGTCGGAGCGGAATGT
SGK1-R	TGTCAGCAGTCTGGAAAGAGA
TIPARP-F	AGAACGAGTGGTTCCAATCCA
TIPARP-R	TGGGTGCAAAAGATCAGTCTG
RASD1-F	AGCTGAGTATCCCGGCCAA
RASD1-R	CGATGGTAGGCGTGTAGGC
BCL6-F	GCCGATGGGATTGAGTGACTGGC
BCL6-R	TGTCTTCACCAATGCCTTGCTTCAC
NFKB1A-F	CTCCGAGACTTTCGAGGAAATAC
NFKB1A-R	GCCATTGTAGTTGGTAGCCTTCA
PPP1R1B-F	GAGCCTCAGCTGGAGATCCG
PPP1R1B-R	TTCGACTTGAGATGGTGCCC
MT1X-F	TCCTTGCCTCGAAATGGACC
MT1X-R	AGGAGCAGCAGCTCTTCTTG
AMOT-F	TTCAAGGGCATGCCACCCCAATC
AMOT-R	CGCTGGCCTGGCTGCTCCATA
